# Plasma Proteomic Profiling Reveals ITGA2B as A Key Regulator of Heart Health in High-altitude Settlers

**DOI:** 10.1093/gpbjnl/qzaf030

**Published:** 2025-04-08

**Authors:** Yihao Wang, Pan Shen, Zhenhui Wu, Bodan Tu, Cheng Zhang, Yongqiang Zhou, Yisi Liu, Guibin Wang, Zhijie Bai, Xianglin Tang, Chengcai Lai, Haitao Lu, Wei Zhou, Yue Gao

**Affiliations:** Beijing Institute of Radiation Medicine, Beijing 100850, China; Beijing Institute of Radiation Medicine, Beijing 100850, China; Beijing Institute of Radiation Medicine, Beijing 100850, China; School of Pharmacy, Jiangxi University of Chinese Medicine, Nanchang 330000, China; Beijing Institute of Radiation Medicine, Beijing 100850, China; School of Pharmacy, Jiangxi University of Chinese Medicine, Nanchang 330000, China; Beijing Institute of Radiation Medicine, Beijing 100850, China; School of Pharmacy, Guangdong Pharmaceutical University, Guangzhou 510006, China; Beijing Institute of Radiation Medicine, Beijing 100850, China; School of Nursing, Capital Medical University, Beijing 100069, China; State Key Laboratory of Medical Proteomics, Beijing Proteome Research Center, National Center for Protein Sciences (Beijing), Beijing Institute of Lifeomics, Beijing 102206, China; Beijing Institute of Radiation Medicine, Beijing 100850, China; Beijing Institute of Radiation Medicine, Beijing 100850, China; Beijing Institute of Radiation Medicine, Beijing 100850, China; School of Chinese Medicine, Hong Kong Traditional Chinese Medicine Phenome Research Center, Hong Kong Baptist University, Hong Kong Special Administrative Region 999077, China; Key Laboratory of Systems Biomedicine (Ministry of Education), Shanghai Center for Systems Biomedicine, State Key Laboratory of Medical Genomics, Ruijin Hospital, Shanghai Jiao Tong University, Shanghai 200240, China; Beijing Institute of Radiation Medicine, Beijing 100850, China; Beijing Institute of Radiation Medicine, Beijing 100850, China; School of Pharmacy, Jiangxi University of Chinese Medicine, Nanchang 330000, China; School of Pharmacy, Guangdong Pharmaceutical University, Guangzhou 510006, China; Department of Nephrology, First Medical Center of Chinese PLA General Hospital, Nephrology Institute of the Chinese People’s Liberation Army, National Key Laboratory of Kidney Diseases, National Clinical Research Center for Kidney Diseases, Beijing Key Laboratory of Kidney Disease Research, Beijing 100853, China

**Keywords:** Myocardial injury, Plasma proteome, ITGA2B, TanⅡA, High-altitude settler

## Abstract

Myocardial injury is a common disease in the plateau, especially in the lowlanders who have migrated to the plateau, in which the pathogenesis is not well understood. Here, we established a cohort of lowlanders comprising individuals from both low-altitude and high-altitude areas and conducted plasma proteomic profiling. Proteomic data showed that there was a significant shift in energy metabolism and inflammatory response in individuals with myocardial abnormalities at high altitude. Notably, integrin alpha-Ⅱb (ITGA2B) emerged as a potential key player in this context. Functional studies demonstrated that ITGA2B upregulated the transcription and secretion of interleukin-6 (IL-6) through the integrin-linked kinase (ILK)/nuclear factor-κB (NF-κB) signaling axis under hypoxic conditions. Moreover, ITGA2B disrupted mitochondrial structure and function, increased glycolytic capacity, and aggravated energy reprogramming from oxidative phosphorylation to glycolysis. Leveraging the therapeutic potential of traditional Chinese medicine in cardiac diseases, we discovered that tanshinone ⅡA (TanⅡA) effectively alleviated the myocardial injury caused by the abnormally elevated expression of ITGA2B and hypobaric hypoxia exposure in mice, thus providing a novel candidate therapeutic strategy for the prevention and treatment of high-altitude myocardial injury.

## Graphical abstract

**Figure:**
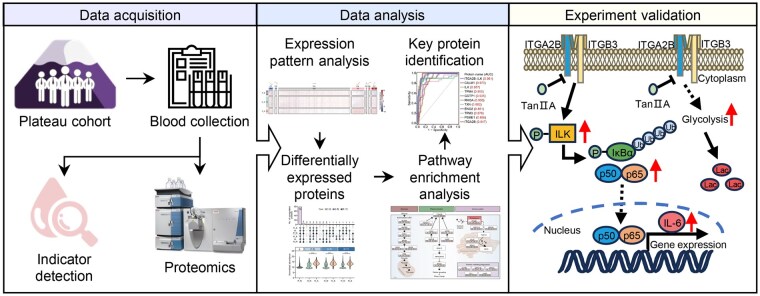

## Introduction

It is widely recognized that people differ in the adaptability to high-altitude environments [1–4]. Chronic mountain sickness (CMS) such as high-altitude polycythemia [5], high-altitude heart disease, and high-altitude pulmonary hypertension [6] will occur if the migrant population cannot adapt well to the hypoxic environments. CMS affects people’s quality of life in highland areas if the symptoms are mild, and in severe cases, it can lead to organ failure and death [7]. The regenerative capacity of the heart for adult mammals is limited which represents an important cause of severe cardiomyopathy and high mortality of heart disease [8,9]. The damage to myocardial cells induced by hypobaric hypoxia often leads to irreversible dysfunction of the heart. However, there is a significant gap in our understanding of the risk factors and early signaling pathways of myocardial injury at high altitude, and an even wider gap in finding protective strategies to mitigate hypoxia-induced myocardial injury.

The integrin members are widely expressed on the cell membrane, and each is made up of one α and one β subunit [10]. Integrin signals can be transmitted by integrin-linked kinase (ILK), which mediates reparative dysfunction in myocardial repair and participates in regulating the transcription and secretion of inflammatory factors by activating nuclear factor-κB (NF-κB) [11]. Studies have shown that integrin expression in cardiomyocytes is associated with inflammatory response [12,13]. Meanwhile, integrin signaling could mediate the adaptive response in the face of hypoxia, such as the shift of energy metabolism from oxidative phosphorylation to glycolysis [14,15]. The main integrin heterodimers in cardiomyocytes are α5β1 and αvβ3 [16]. Importantly, integrin alpha-Ⅱb (αⅡb, ITGA2B), a chaperone of integrin β3, is primarily expressed in platelets [17] and also present in small amounts in the heart [18,19]. Several studies in recent years have shown that ITGA2B is an important predictor of cardiovascular disease. ITGA2B was highly expressed in the peripheral blood of patients suffering from pulmonary hypertension, proposing that ITGA2B is the hub protein of pulmonary hypertension [20]. ITGA2B was also identified as a potentially upregulated protein in the development of heart failure after ST-segment elevation myocardial infarction [21]. The content of ITGA2B in peripheral blood and its gene polymorphisms are closely related to the severity of cardiovascular disease [22,23]. These findings suggest that ITGA2B mediates cardiac disease progression through an unidentified mechanism, and has the potential to serve as an important driver of myocardial injury induced by high altitude.

Content changes of some plasma proteins have been widely recognized as indicators of pathophysiological changes induced by various diseases. It is reported that plasma proteome can reflect the physiological condition of the cardiovascular system [24], a fact that has been utilized for decades in the routine analysis of plasma biomarkers for the purpose of diagnosing and monitoring of cardiovascular diseases. For example, creatine kinase-MB isoenzyme (CK-MB) in plasma is an important molecular marker of myocardial injury caused by chronic hypoxia [25], which plays a central role in energy transduction in heart tissue. Proteomics technology provides a new strategy for the analysis of plasma proteins, which greatly promotes the discovery of risk biomarkers [26], the analysis of pathogenesis, and the study of drug action mechanisms [27] of high-altitude diseases. However, no studies have yet investigated the plasma proteomic changes in high-altitude settlers with myocardial abnormalities. Therefore, a detailed description of the plasma proteomic characteristics of the population with high-altitude myocardial abnormalities will provide important information for the pathogenesis of this disease.

Tanshinone ⅡA (TanⅡA) is a flavonoid compound discovered from the traditional Chinese herb *Salvia miltiorrhiza* Bunge. Tanshinone sodium ⅡA sulfonate injection with TanⅡA as the main component has been widely used in cardiovascular diseases. Although our research group’s previous study has demonstrated that TanⅡA has a significantly enhanced therapeutic effect on high-altitude diseases, there are few reports on myocardial injury at high altitude.

In this study, we conducted data-independent acquisition (DIA) proteomics on the plasma samples from native lowlanders who developed myocardial abnormalities after migrating to the plateau, aiming to dig the molecular mechanism of myocardial injury caused by chronic hypobaric hypoxia. Both ITGA2B and ILK proteins were highly expressed in the migrants with myocardial abnormalities, promoting the transcriptional activation of NF-κB and resulting in inflammatory factor production. Meanwhile, we also found that TanⅡA attenuated the high-altitude myocardial abnormalities caused by aberrant ITGA2B overexpression (OE). These findings not only provide new ideas for the early intervention of high-altitude heart disease, but also identify a promising candidate drug for the treatment of high-altitude myocardial injury.

## Results

### Plasma proteomic analysis of migrants with myocardial abnormalities at high altitude

**Figure 1:**
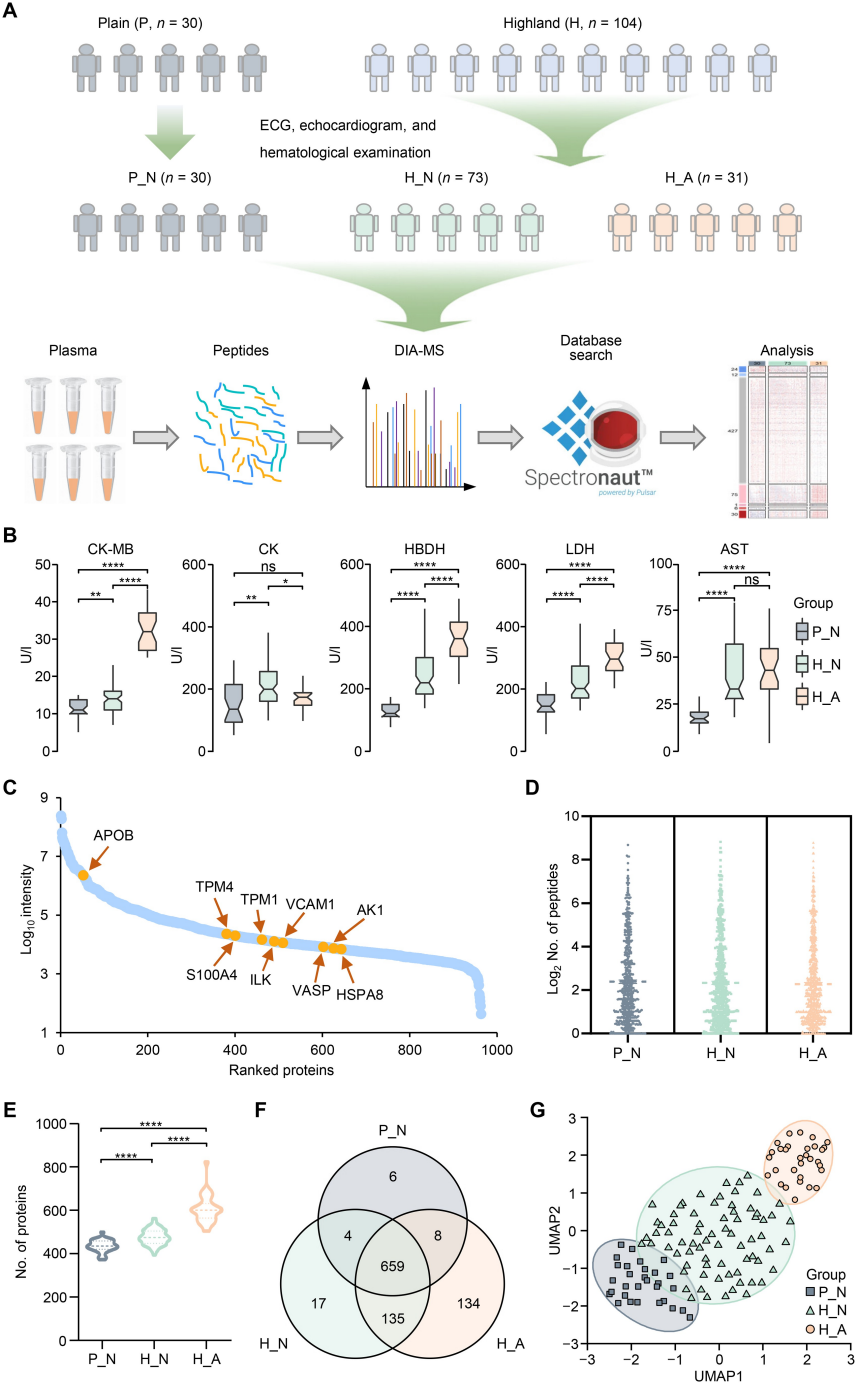
Overview of DIA proteomic profiling of plasma in plain and highland individuals **A**. Flowchart to depict the plasma proteome profile of high-altitude settlers. The study cohort includes 30 plain individuals (P_N) and 104 migrants whose BMI and age distributions are similar (Table S1). Based on the results of hematological and imaging tests, the plateau migrants are divided into cardiac normal (H_N) and cardiac abnormal (H_A) groups. Finally, DIA plasma proteomic profiling was performed. **B**. Results of myocardial enzyme spectrum in the peripheral blood. *, *P* < 0.05; **, *P* < 0.01; ****, *P* < 0.0001; ns, not significant (Mann-Whitney U test). **C**. Dynamic range of protein quantification intensity in 134 plasma samples. Several common blood biomarkers and heart-related proteins are marked. **D**. Distribution of the number of peptides corresponding to each identified protein in three groups of plasma samples. **E**. Number of identified proteins in each plasma sample from three groups. ****, *P* < 0.0001 (one-way ANOVA). **F**. Venn plot of proteins identified from plasma samples in P_N, H_N, and H_A, respectively. **G**. UMAP for dimension reduction showing the overall difference among the three groups. The optimal enclosing ellipses at tolerance level of 0.01 were estimated using the Khachiyan algorithm. DIA, data-independent acquisition; BMI, body mass index; MS, mass spectrometry; UMAP, uniform manifold approximation and projection; ANOVA, analysis of variance; ECG, electrocardiogram; CK-MB, creatine kinase-MB; CK, creatine kinase; HBDH, hydroxybutyrate dehydrogenase; LDH, lactate dehydrogenase; AST, alanine aminotransferase.

We recruited individuals born and raised in low-altitude areas from Chengdu, Sichuan Province, China (P_N, *n* = 30) and Xizang Autonomous Region, China (*n* = 104). Individuals who migrated to Xizang Autonomous Region, China were further divided into high-altitude myocardial normal (H_N, CK-MB ≤ 25 U/l, *n* = 73) and high-altitude myocardial abnormal (H_A, CK-MB > 25 U/l, *n* = 31) groups based on serum CK-MB levels. Detailed clinical characteristics, including age, body mass index (BMI), important indicators of blood routine test, and duration of migration to plateau, are shown in Figure S1A and Table S1. There were no significant differences in BMI and age distributions among the three groups. Therefore, we used this cohort to establish a comprehensive plasma proteome profile of high-altitude myocardial injury (**Figure 1**A). The blood oxygen saturation of the H_N and H_A groups was notably lower than that in the P_N group (Figure S1B). Correspondingly, the red blood cell (RBC) count and hemoglobin (HGB) level were significantly higher in the H_N and H_A groups, while the platelet (PLT) count was lower than that in the P_N group (Figure S1C). Moreover, dyslipidemia occurred in both the H_N and H_A groups (Figure S1D); however, no significant differences were observed between these two groups in oxygen saturation, routine blood results, and lipid content. The main difference between the H_A and H_N groups was in the content of myocardial enzymes, such as, CK-MB, lactate dehydrogenase (LDH), and hydroxybutyrate dehydrogenase (HBDH), which were significantly increased in the H_A group (Figure 1B). Additionally, while the migration duration of H_A was slightly longer than that of H_N, this difference was not statistically significant (Figure S1A).

A total of 963 protein groups were identified from 134 plasma samples, and the protein quantification information spanned 7 orders of magnitude (Figure 1C). The average number of identified peptides per protein was 5.4 (Figure 1D, Figure S1E). More than 80% of the proteins had *P* < 0.01 (Figure S1F), and almost all of the identified proteins had C-score > 0.8, with high scores indicating high-quality identifications (Figure S1G). These results indicated that the quality of the dataset was reliable. The average number of identified proteins per sample gradually increased across the three groups: P_N (*n* = 441 proteins), H_N (*n* = 481 proteins), and H_A (*n* = 608 proteins) (Figure 1E). The Venn plot showed that 659 proteins were identified by three groups, and 134 proteins were uniquely identified in H_A, which was the largest among the three groups (Figure 1F). Then, we found that the plasma proteomic characteristics of the three groups were clearly different (Figure 1G) through uniform manifold approximation and projection (UMAP) analysis. Correlation analysis also revealed that the plasma proteomic characteristics of H_A showed the greatest deviation from that of P_N (Figure S1H). These results indicate that myocardial injury caused by chronic hypoxia could cause significant perturbation of the plasma proteome.

### Proteomic signatures in plasma of the H_A group

To quantitatively compare protein expression differences among the three groups, 575 proteins were selected based on the proportion of missing values of quantitative information (< 25%). Differential expression analysis revealed 61 differentially expressed proteins (DEPs) between H_N and P_N, 118 DEPs between H_A and H_N, and 193 DEPs between H_A and P_N (Figure S2A), and most of these DEPs were upregulated. Functional enrichment analysis of these upregulated DEPs also showed that the enrichment significance of platelet activation, glycolytic process, oxidative stress, and carbon metabolism gradually increased between pairwise comparisons: H_N *vs*. P_N < H_A *vs*. H_N < H_A *vs*. P_N (Figure S2B), further indicating the heart-related dysfunction in the H_A group. Plasma proteomic changes in H_A highlighted the important role of upregulated DEPs in the process of myocardial injury at high altitude.

**Figure 2:**
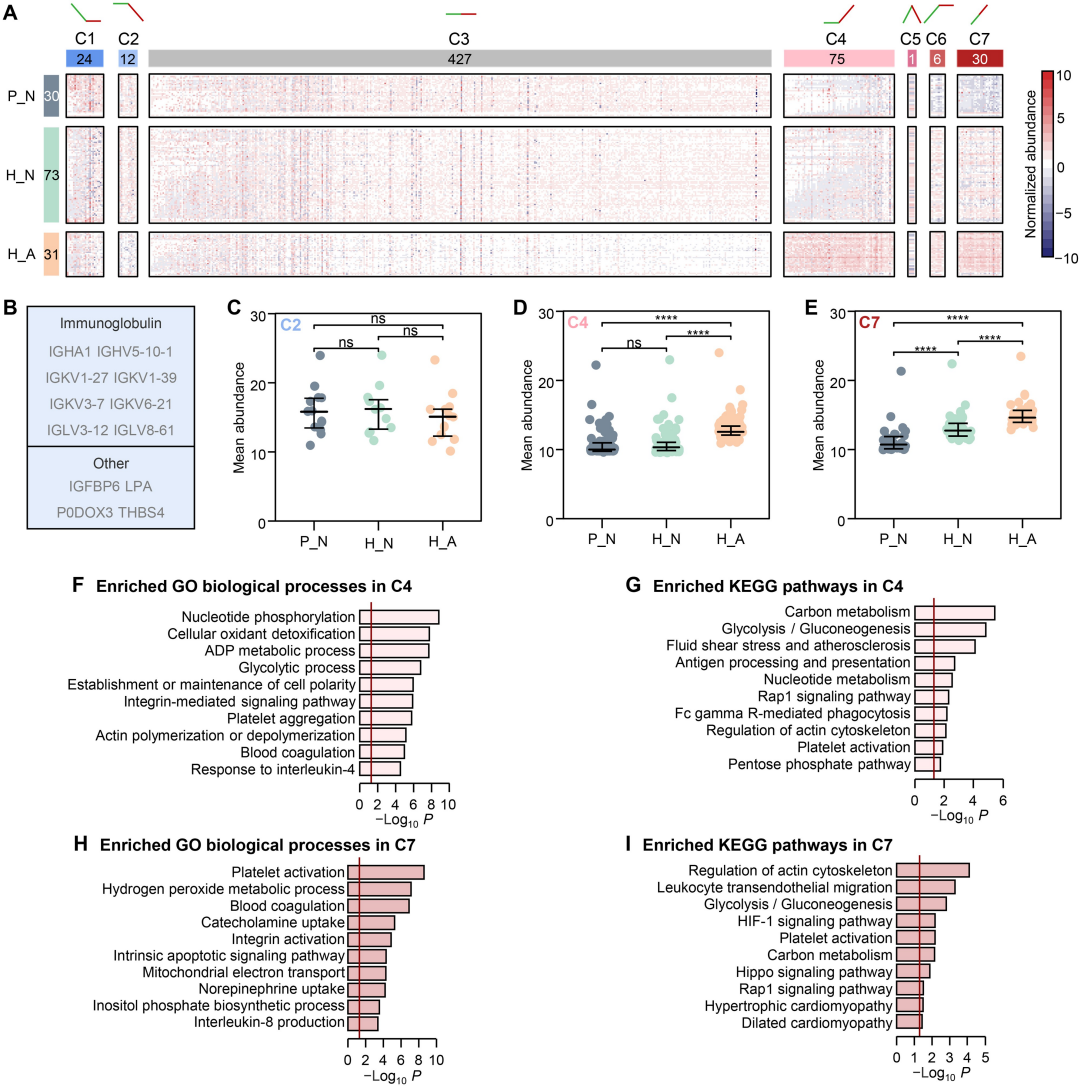
Obvious differences in protein abundance were found among the P_N, H_N, and H_A datasets **A**. Heatmap showing protein abundance in seven clusters in P_N, H_N, and H_A datasets. The expression trends of clusters are represented by green (H_N *vs.* P_N) and red (H_A *vs.* H_N) lines at the top of the heatmap. Numbers in the left boxes denote the number of samples in each population. Numbers in the boxes above the heatmap indicate the number of proteins in each cluster. **B**. The proteins classified in C2. **C**. Mean protein abundance of C2 in three datasets. **D**. Mean protein abundance of C4 in three datasets. **E**. Mean protein abundance of C7 in three datasets. In (C–E), ****, *P* < 0.0001; ns, not significant (Mann-Whitney U test). **F**. Enriched GO biological processes in C4. **G**. Enriched KEGG pathways in C4. **H**. Enriched GO biological processes in C7. **I**. Enriched KEGG pathways in C7. In (F–I), the red lines represent −log_10_ 0.5.GO, Gene Ontology; KEGG, Kyoto Encyclopedia of Genes and Genomes.

By analyzing the Venn diagram of DEPs among the three groups (Figure S2C) and their regulation patterns (up and down) (Figure S2D), the 575 selected proteins were divided into 7 clusters (**Figure 2**A). To identify proteins specifically dysregulated in the H_A population, we focused on Cluster 2 (C2, *n* = 12), Cluster 4 (C4, *n* = 75), and Cluster 7 (C7, *n* = 30), in which proteins displayed upregulated or downregulated trends in the H_A group (Table S2). The proteins in C2, primarily composed of immunoglobulins (Figure 2B), showed a specific downregulation in H_A compared to H_N (Table S2). However, due to the relatively small number of proteins in C2, the overall comparison shown in Figure 2C did not reach statistical significance. All 30 proteins in C7 were all significantly upregulated, overlapping in pairwise comparisons between the three groups. In C4, 75 proteins were divided into two groups, of which 67 proteins were upregulated in both H_A *vs*. H_N and H_A *vs*. P_N comparisons, and 8 proteins were only upregulated in H_A *vs*. H_N comparison. Moreover, the average expression levels of proteins in the H_A group were significantly higher than those in the P_N and H_N groups (Figure 2D and E), suggesting that proteins involved in high-altitude myocardial injury might have an upregulated expression pattern. Functional enrichment analysis showed that proteins in C4 and C7 were enriched in energy metabolism, inflammation, and blood coagulation pathways (Figure 2F–I), such as glycolytic process, integrin-mediated signaling pathway, blood coagulation, and mitochondrial electron transport. These functions are closely related to myocardial injury, which further suggests that the proteins with upregulation trends may play an important role in myocardial injury at high altitude.

### Correlation analysis of upregulated DEPs with residence duration and myocardial enzymes

To explore the effect of migration duration on myocardial injury at high altitude, we divided the duration of plateau settlement into three phases: 1–3 years, 4–5 years, and 6–11 years. CK-MB content increased progressively during the first five years, and plateaued at 6–11 years (Figure S3A). The proportion of CK-MB abnormality (CK-MB > 25 U/l) showed a similar trend, which progressively increased during the first five years, while leveled off at 6–11 years (Figure S3B).

**Figure 3:**
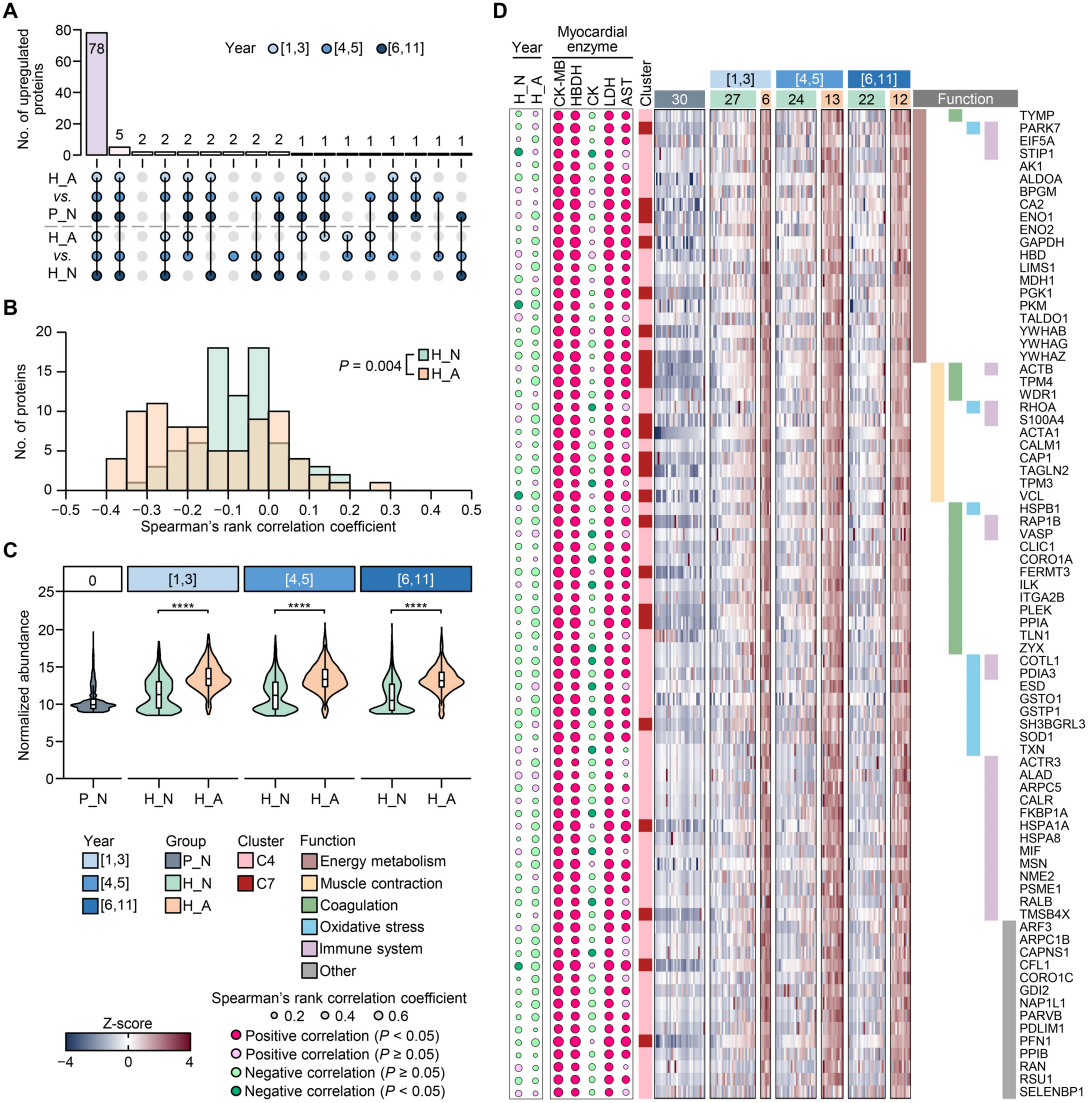
The dysregulated proteins in H_A were multifunctional **A**. Upset plot showing the number of upregulated proteins in different categories. **B**. Distribution of the 78 upregulated proteins showing distinct correlations with residence duration in H_N and H_A. Spearman’s rank correlation coefficients were calculated. *P* value between H_N and H_A was calculated by Mann-Whitney U test. **C**. Violin plots showing protein abundance in P_N, H_N, and H_A across different residence durations. ****, *P* < 0.0001 (Mann-Whitney U test). **D**. Heatmap showing the abundance of various functional proteins. Spearman’s rank correlation coefficients between the residence duration (year), myocardial enzyme activity, and abundance of each protein are shown on the left of the heatmap.

It was found that the vast majority of proteins (78/105, 74.3%) in C4 and C7 were significantly different in each duration category (**Figure 3**A). Spearman correlation analysis showed weak negative correlations between residence duration and the expression levels of these 78 proteins, especially in the H_A group (Figure 3B). The intact proteomic data indicated that the features of H_N in 6–11 years were more inclined to P_N, and the corresponding proteomic features of H_A in 6–11 years were more inclined to H_N (Figure S3C). These results indicate that the abundance of the abnormally highly expressed proteins in H_A may decrease sightly with increasing residence time (Figure 3C). Functional enrichment analysis showed that most of the 78 proteins were enriched in energy metabolism, muscle contraction, coagulation, oxidative stress, and immunity-related processes (Figure 3D). Interestingly, CK-MB had an obviously positive correlation with several key proteins in glycolysis and tricarboxylic acid cycle, such as BPGM, MDH, PKM, and GAPDH. In addition, CK-MB also showed a positive correlation with CA2 and HBD, which play important roles in regulating the body’s gas exchange.

These results suggest that the upregulated proteins in populations with high-altitude myocardial injury already exhibit increased expression levels during the initial stages of migration. As the duration of migration increases, there may be a slight decrease in expression levels, potentially due to adaptive regulation by the body. However, given the lack of precise timing of myocardial abnormalities in these populations, it is difficult to robustly demonstrate the trend of changes in these proteins with abnormal expression during the occurrence and progression of the myocardial injury.

### Screening and validation of key proteins in high-altitude myocardial injury

**Figure 4:**
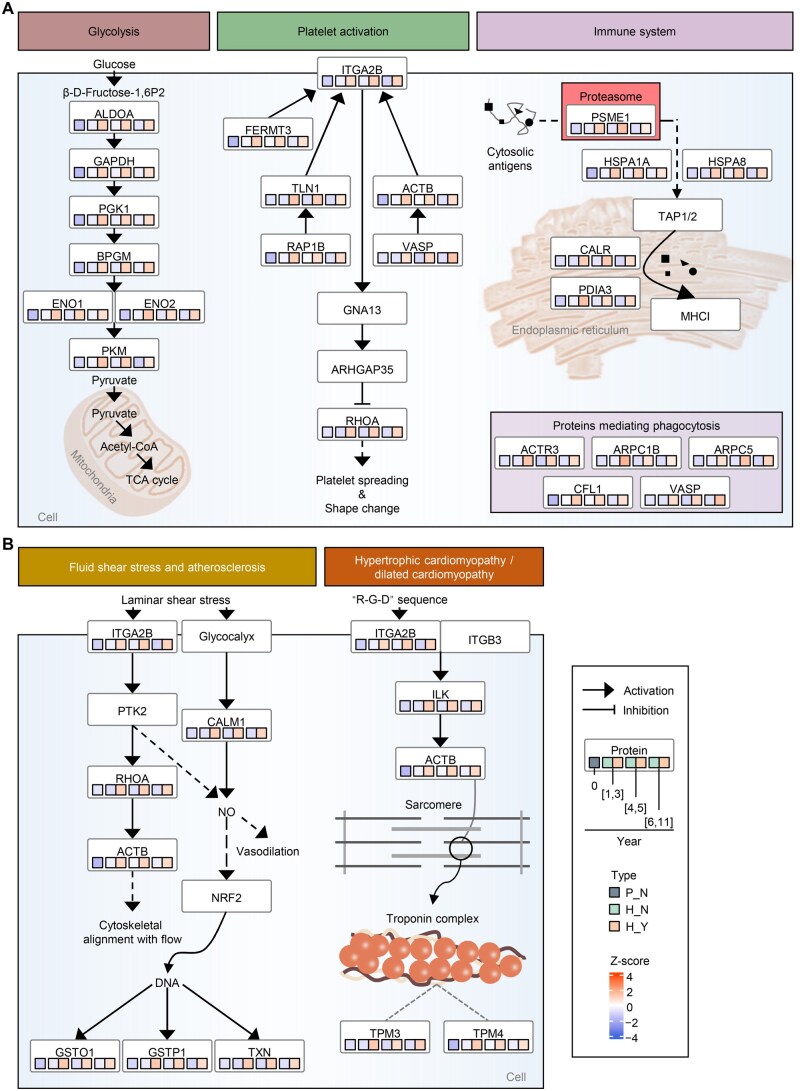
The dysregulated proteins in key pathways implicated in myocardial injury were affected by highland Abnormally high expression of ITGA2B in H_A could cause platelet shape change and degranulation, mediate cytoskeletal alignment with flow through RHOA, and conduct integrin signaling via ILK kinase. Key proteins in different categories in multiple normal (**A**) and disease (**B**) pathways are indicated with their corresponding expression levels in three groups across different residence durations. ITGA2B, integrin alpha-Ⅱb; ILK, integrin-linked kinase.

Pathways enriched with abnormally expressed proteins related to myocardial injury attracted our attention, including three normal physiological processes (glycolysis, platelet activation, and immune system) (**Figure 4**A) and two disease-associated pathways (fluid shear stress and atherosclerosis, and hypertrophic cardiomyopathy/dilated cardiomyopathy) (Figure 4B). Most of the perturbations in protein abundance originated from chronic hypoxia exposure, with DEPs in H_A typically showing increased abundance compared to those in H_N.

**Figure 5:**
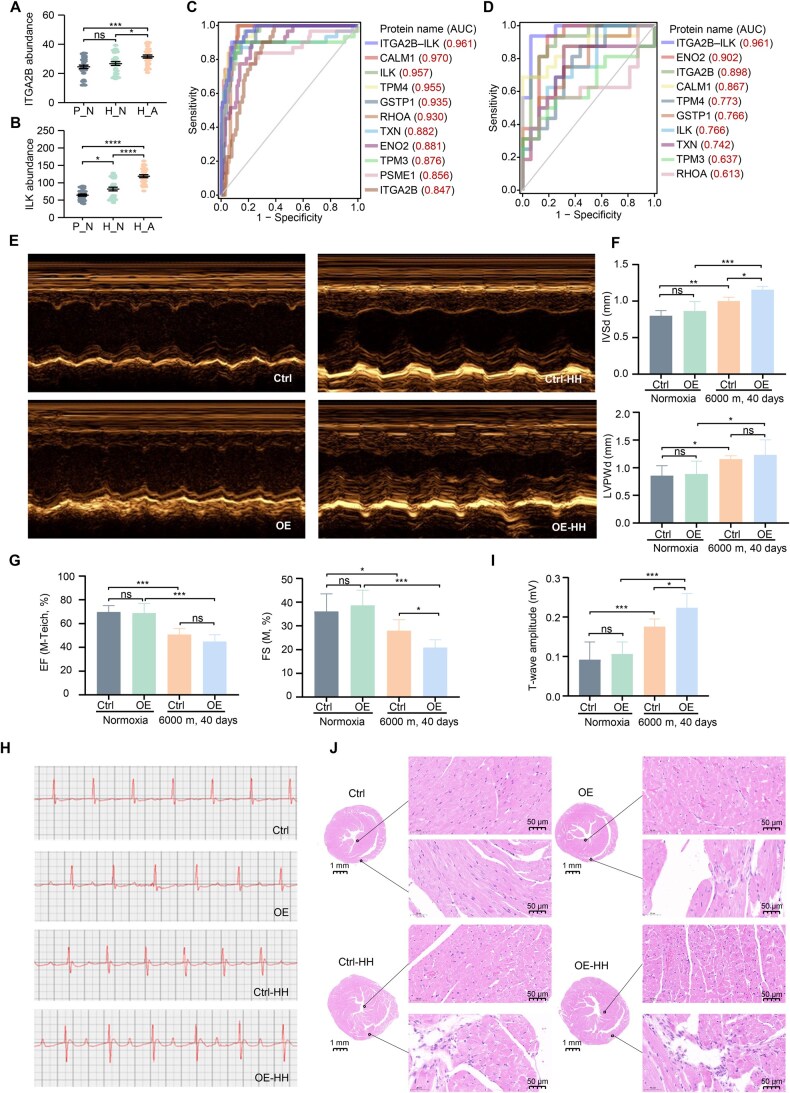
ITGA2B OE aggravated the structural and functional damage of mouse hearts under hypobaric hypoxia exposure **A**. ITGA2B abundance in the P_N, H_N, and H_A groups measured by ELISA. *, *P* < 0.05; ***, *P* < 0.001; ns, not significant (one-way ANOVA). **B**. ILK abundance in the P_N, H_N, and H_A groups measured by ELISA. *, *P* < 0.05; ****, *P* < 0.0001 (one-way ANOVA). **C**. Evaluation of the classification performance of the 10 key DEPs and the ITGA2B–ILK signaling axis in distinguishing between H_N and H_A groups. **D**. Verification of the classification performance of the 10 key DEPs and the ITGA2B–ILK signaling axis in an independent cohort using PRM proteomic data. **E**. Representative echocardiographic images of mice overexpressing ITGA2B-FLAG or FLAG under normoxic or hypobaric hypoxic conditions. “Ctrl” refers to mice overexpressing FLAG under normoxic conditions; “OE” refers to mice overexpressing ITGA2B-FLAG under normoxic conditions; “Ctrl-HH” refers to mice overexpressing FLAG under hypobaric hypoxic conditions; and “OE-HH” refers to mice overexpressing ITGA2B-FLAG under hypobaric hypoxic conditions. **F**. Bar plots showing the IVSd (top) and LVPWd (bottom) of mice overexpressing ITGA2B-FLAG or FLAG under normoxic or hypobaric hypoxic conditions. Data are represented as mean ± SD (*n* = 6). *, *P* < 0.05; **, *P* < 0.01; ***, *P* < 0.001; ns, not significant (one-way ANOVA). **G**. Bar plots showing the EF (left) and FS (right) of mice overexpressing ITGA2B-FLAG or FLAG under normoxic or hypobaric hypoxic conditions. Data are represented as mean ± SD (*n* = 6). *, *P* < 0.05; ***, *P* < 0.001; ns, not significant (one-way ANOVA). **H**. Representative ECG images of mice overexpressing ITGA2B-FLAG or FLAG under normoxic or hypobaric hypoxic conditions. **I**. Changes in T-wave amplitude in the ECG of mice overexpressing ITGA2B-FLAG or FLAG under normoxic or hypobaric hypoxic conditions. Data are represented as mean ± SD (*n* = 6). *, *P* < 0.05; ***, *P* < 0.001; ns, not significant (one-way ANOVA). **J**. Representative H&E staining images displaying the pathological structural changes of heart tissues in different groups of mice. ELISA, enzyme-linked immunosorbent assay; DEP, differentially expressed protein; PRM, parallel reaction monitoring; AUC, area under the curve; OE, overexpression; HH, hypobaric hypoxia; IVSd, interventricular septal end-diastolic dimension; LVPWd, left ventricular end-diastolic posterior wall dimension; EF, ejection fraction; FS, left ventricular fractional shortening; SD, standard deviation; H&E, hematoxylin and eosin.

In the aforementioned pathways, a total of 10 DEPs were identified, including CALM1, ENO2, GSTP1, ITGA2B, ILK, PSME1, RHOA, TPM3, TPM4, and TXN. All the 10 DEPs showed significant abundance differences in different residence durations (Figure S3D and E), consistent with differential expression analysis. As verified by enzyme-linked immunosorbent assay (ELISA) (Figure S3F and G), only ENO2 levels showed significant differences between H_A and H_N among all three residence durations, indicating that the increase in the glycolysis process was further aggravated in the H_A group. In addition,GSTP1, RHOA, and TXN levels were significantly upregulated in two phases, indicating that severe oxidative stress might also exist in H_A individuals. However, ITGA2B levels showed no statistically significant differences among the three residence durations. Notably, regardless of the residence duration effects, the levels of ITGA2B and its downstream effector ILK were significantly increased in H_A (**Figure 5**A and B). ITGA2B is involved in a variety of pathophysiological processes, including platelet activation, blood pressure, and hypertrophic cardiomyopathy.

We constructed prediction models for H_N and H_A populations using the aforementioned DEPs and the ITGA2B–ILK signaling axis (Figure 5C), and found that this signaling axis exhibited exceptional classification performance, achieving an area under the curve (AUC) of 0.961. To further verify the discriminative power of ITGA2B–ILK, an independent cohort (16 H_N and 16 H_A individuals) was established. There were no significant differences in their age, BMI, and time of residence at the plateau, but the myocardial enzyme profile of H_A population was significantly abnormal. Parallel reaction monitoring (PRM) collection mode was used to target the 10 key DEPs. We used the quantitative results of these proteins to verify the classification model, and the results showed that the ITGA2B–ILK signaling axis had the best classification effect (Figure 5D). Therefore, we then focused on the molecular mechanism of the ITGA2B–ILK axis in high-altitude myocardial injury.

### ITGA2B OE aggravated the cardiac damage caused by long-term hypoxia

AC16 cardiomyocytes stably overexpressing ITGA2B-FLAG and green fluorescent protein (GFP) were constructed using lentiviral vectors, which were confirmed by fluorescence photographs (Figure S4A), real-time quantitative reverse transcription polymerase chain reaction (RT-qPCR) (Figure S4B, left), and Western blot (WB) (Figure S4C, left). Mice overexpressing ITGA2B were generated by *in situ* injection of an adeno-associated virus serotype 9 (AAV9) vector carrying the cardiac troponin I (*CTNI*) promoter, which were confirmed by RT-qPCR (Figure S4B, right) and WB (Figure S4C, right). In addition, we performed proteomic analysis on AC16 cells (Figure S4D) and found that ITGA2B protein was highly expressed in OE AC16 cells (Figure S4E). ELISA results also showed that ITGA2B was highly expressed in OE mouse hearts (Figure S4F).

We first investigated the effects of ITGA2B OE on cardiac structure and function. Echocardiography in mice was not altered by ITGA2B OE under normoxic conditions (Ctrl *vs.* OE; Figure 5E). However, after 40 days of hypobaric hypoxia exposure (HH; 6000 m), significant changes in echocardiography were observed (Ctrl-HH *vs.* Ctrl and OE-HH *vs.* OE; Figure 5E). The interventricular septal end-diastolic dimension (IVSd) and left ventricular end-diastolic posterior wall dimension (LVPWd) were significantly thickened (Ctrl-HH *vs.* Ctrl; Figure 5F), and ITGA2B OE caused further thickening of IVSd under hypobaric hypoxia (OE-HH *vs.* Ctrl-HH; Figure 5F). ITGA2B OE also exacerbated the reduction in the fractional shortening of the short axis in mouse hearts (OE-HH *vs.* Ctrl-HH; Figure 5G). The electrocardiogram (ECG) results indicated an increase in T-wave amplitude in Ctrl-HH mice compared to Ctrl mice and an even greater increase in the T-wave amplitude in OE-HH mice (Figure 5H and I). These results suggest that ITGA2B could cause cardiac dysfunction under hypobaric hypoxic conditions and further aggravate cardiac hypertrophy.

We also observed that the hearts of OE-HH mice became darker and enlarged (Figure S5A), and the organ coefficient of the heart was significantly increased (Figure S5B). The hematoxylin and eosin (H&E) staining results showed that the papillary muscle cells were enlarged, microvessels were increased, microthrombus appeared, and inflammatory cell infiltration occurred in the right ventricle in OE-HH mice (Figure 5J). Larger hearts and obvious fibrotic lesions in the right ventricle were observed in OE-HH mice by Masson staining (Figure S5C). Parallelly, ITGA2B OE also resulted in significant increases in CK-MB and NT-proBNP levels in the serum of mice (Figure S4G and H). These results indicated that ITGA2B OE aggravated cardiac structural and functional damage in mice under hypobaric hypoxic conditions.

ITGA2B OE also led to a significant increase in CK-MB secretion in AC16 cells under hypoxic conditions (1% O_2_), termed OE-H AC16 cells (Figure S4I). We found that the levels of HBDH, LDH, and CTNI were significantly increased in the culture medium of OE-H AC16 cells (Figure S5D). The flow cytometric phenotype of OE-H AC16 cells was also changed (Figure S5E), with a significant increase in intracellular granularity (Figure S5F). The proteomic data of AC16 cells also indicated that ITGA2B mainly affected the proteomic profile of AC16 cells under hypoxic conditions (Figure S5G). The Ctrl-H and OE-H AC16 cells were clearly distinguished in principal component analysis (PCA). These results suggest that ITGA2B OE has no obvious effect on cardiomyocytes under normoxia, but significantly alters the phenotype of cardiomyocytes under hypoxia.

### ITGA2B OE aggravated the inflammatory response and energy metabolism disorders

**Figure 6:**
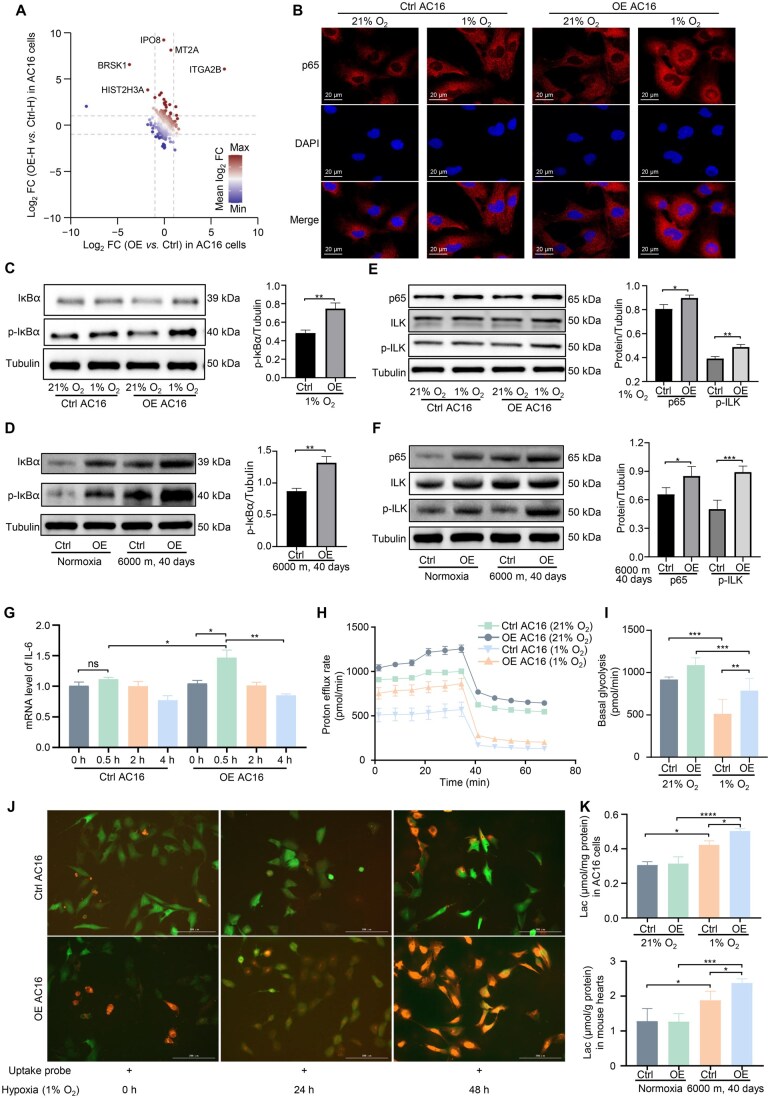
ITGA2B OE exacerbated hypoxia-induced inflammatory responses and energy metabolism disorders **A**. Screening DEPs in OE AC16 cells after hypoxia exposure using the proteomic data of AC16 cells to clarify the biological processes affected by ITGA2B. “Ctrl” refers to AC16 cells overexpressing GFP/FLAG under normoxic conditions (21% O_2_); “OE” refers to AC16 cells overexpressing ITGA2B-FLAG under normoxic conditions (21% O_2_); “Ctrl-H” refers to AC16 cells overexpressing GFP/FLAG under hypoxic conditions (1% O_2_); “OE-H” refers to AC16 cells overexpressing ITGA2B-FLAG under hypoxic conditions (1% O_2_). **B**. Confocal microscopy showing selective upregulation of nuclear NF-κB p65 in the OE AC16 cells after hypoxia exposure (1% O_2_, 24 h). **C**. and **D**. WB assays of the expression levels of IκBα and p-IκBα in Ctrl and OE AC16 cells (C) and mouse hearts (D) with or without hypoxia exposure. **, *P* < 0.01 (Student’s *t*-test). **E**. and **F**. WB assays of the levels of NF-κB p65, ILK, and p-ILK in Ctrl and OE AC16 cells (E) and mouse hearts (F) with or without hypoxia exposure. *, *P* < 0.05; **, *P* < 0.01; ***, *P* < 0.001 (Student’s *t*-test). **G**. RT-qPCR analysis showing the mRNA levels of IL-6 in Ctrl and OE AC16 cells with different durations of hypoxia exposure. Data are presented by mean ± SD (*n* = 4). *, *P* < 0.5; **, *P* < 0.01; ns, not significant (Student’s *t*-test). **H**. Measurement of glycolytic activities in Ctrl and OE AC16 cells with or without hypoxia exposure (1% O_2_, 24 h). **I**. Quantification of glycolytic activities shown in (H). **J**. Glucose intake tests indicating the changes in the ability of Ctrl and OE AC16 cells to take up glucose under different durations of hypoxia exposure. Red indicates the signal of glucose analogs. **K**. Content change of Lac in Ctrl and OE AC16 cells (up) and mouse hearts (down) after hypoxia exposure. *, *P* < 0.05; ***, *P* < 0.001; ****, *P* < 0.0001 (one-way ANOVA). FC, fold change; DAPI, 4′,6-diamidino-2-phenylindole; GFP, green fluorescent protein; p-IκBα, phospho-IκBα; p-ILK, phospho-ILK; mRNA, messenger RNA; IL-6, interleukin-6; RT-qPCR, real-time quantitative reverse transcription polymerase chain reaction; Lac, lactic acid; WB, Western blot.

To investigate how ITGA2B exacerbates structural and functional damage in cardiac cells and hearts, we performed comparative proteomic analyses on AC16 cells. The results revealed that the transcription-related proteins IPO8 and HIST2H3A and the NF-κB pathway-related protein MT2A were highly expressed in OE-H AC16 cells compared to Ctrl-H AC16 cells (**Figure 6**A; Table S3). Given the regulation of NF-κB p65 transcriptional activity by integrin–ILK axis, we found that p65 was accumulated in the nucleus in OE-H AC16 cells (Figure 6B). The levels of phospho-IκBα (p-IκBα) were significantly increased in OE-H AC16 cells and OE-HH mouse heart tissues (Figure 6C and D). Furthermore, we found that ITGA2B OE significantly increased the levels of phospho-ILK (p-ILK) and p65 in OE-H AC16 cells and OE-HH mouse heart tissues (Figure 6E and F). Increased NF-κB transcriptional activity was further confirmed by increased interleukin-6 (IL-6) messenger RNA (mRNA) levels during the early hypoxic phase (Figure 6G). However, as the duration of hypoxia increased, the mRNA levels of IL-6 decreased (Figure S6A and B), probably due to the existence of negative feedback. Anyway, ITGA2B OE significantly increased the levels of IL-6 in AC16 cell culture medium (Figure S6C) and mouse heart tissues (Figure S6D) after hypoxia exposure.

Using electron microscopy, we found marked mitochondrial swelling, matrix dissolution within the membrane, and even membrane disruption in OE-H AC16 cells (Figure S6E). Meanwhile, ITGA2B OE exacerbated hypoxia-induced oxidative stress, with increased malondialdehyde (MDA) content in OE-H AC16 cells and OE-HH mouse heart tissues (Figure S6F) and reactive oxygen species (ROS) levels in OE-H AC16 cells (Figure S6G). Considering that glycolysis-related proteins were upregulated in H_A according to the plasma proteomic data described above, we explored whether ITGA2B OE could exacerbate hypoxia-induced increase in glycolysis. A glycolysis rate assay using AC16 cells was performed (Figure 6H). Results showed that ITGA2B OE increased basal glycolysis in AC16 cells (Figure 6I). Furthermore, glucose uptake assay showed that ITGA2B OE led to a significant increase in glucose uptake in AC16 cells after hypoxia exposure (Figure 6J). The changes of lactic acid (Figure 6K) and adenosine triphosphate (ATP) (Figure S6H) contents also indicated that ITGA2B OE aggravated the shift of energy metabolism from oxidative phosphorylation to glycolysis under hypoxic conditions.

### TanⅡA ameliorated cardiac injury caused by ITGA2B OE under hypobaric hypoxia exposure

**Figure 7:**
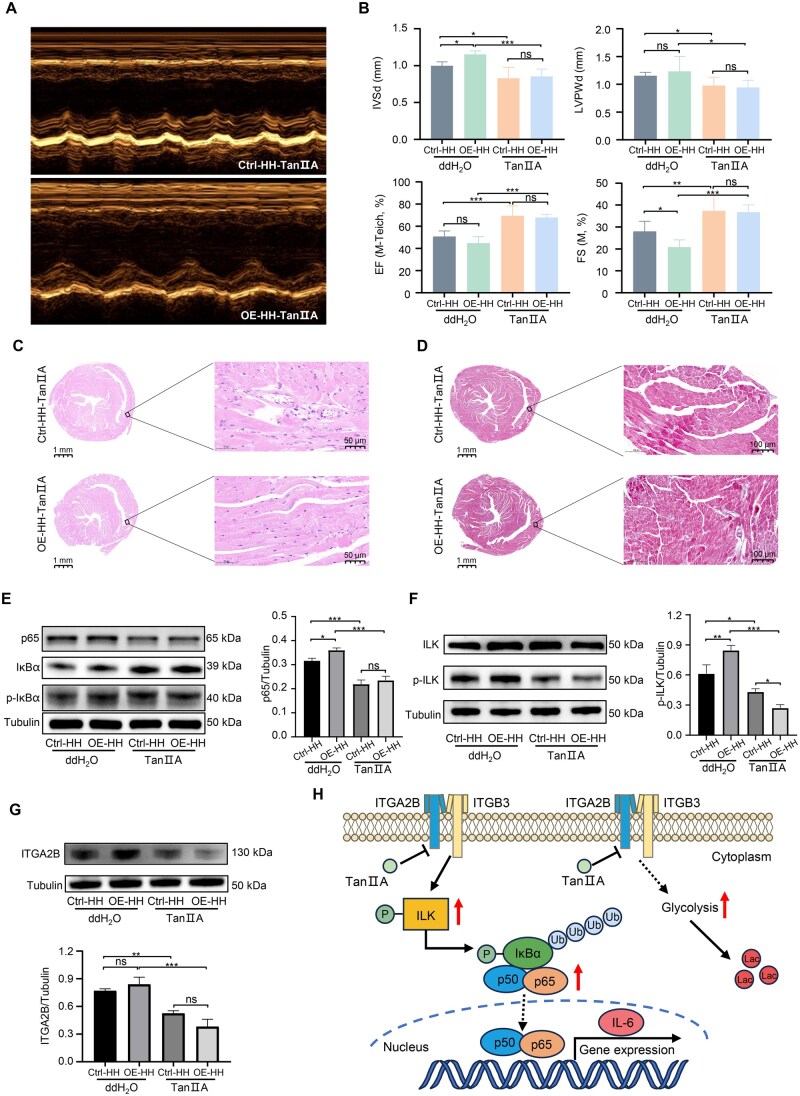
TanⅡA ameliorated cardiac structural and functional damage caused by ITGA2B OE under hypobaric hypoxia exposure **A**. Representative echocardiographic images of Ctrl-HH and OE-HH mice treated with TanⅡA. **B**. TanⅡA reduced the thicknesses of IVSd and LVPWd and improved EF and FS in Ctrl-HH and OE-HH mice. Data are presented as mean ± SD (*n* = 6). *, *P* < 0.05; **, *P* < 0.01; ***, *P* < 0.001; ns, not significant (one-way ANOVA). **C**. H&E staining of heart tissues from the Ctrl-HH and OE-HH mice treated with TanⅡA. Scale bars, 1 mm or 50 μm. **D**. Masson staining of heart tissues from the Ctrl-HH and OE-HH mice treated with TanⅡA. Scale bar, 1 mm or 100 μm. **E**. WB assay of the expression levels of p65, IκBα, and p-IκBα in Ctrl-HH and OE-HH mice with or without TanⅡA treatment. **F**. WB assay of the expression levels of ILK and p-ILK in Ctrl-HH and OE-HH mice with or without TanⅡA treatment. **G**. WB assay of the expression levels of ITGA2B in Ctrl-HH and OE-HH mice with or without TanⅡA treatment. Data are presented as mean ± SD (*n* = 3). **, *P* < 0.01; ***, *P* < 0.001; ns, not significant (one-way ANOVA). **H**. Schematic of the mechanism by which TanⅡA alleviates cardiac injury caused by abnormally high expression of ITGA2B at high altitude. TanⅡA, tanshinone ⅡA; ITGB3, integrin beta-3; Ub, ubiquitin.

Furthermore, we aimed to investigate whether ITGA2B can serve as a potential drug target for intervention of high-altitude myocardial injury. TanⅡA has aroused our interest due to its widespread use in treating cardiovascular diseases [28]. Encouragingly, we observed significant improvements in echocardiography in both OE-HH and Ctrl-HH mice treated with TanⅡA (**Figure 7**A). Specifically, TanⅡA significantly reduced the thicknesses of IVSd and LVPWd, while also increased the ejection fraction and fractional shortening of the mouse hearts in both groups (Figure 7B). H&E staining results showed that the pathological lesions of the hearts in both OE-HH and Ctrl-HH groups were significantly alleviated, such as reduced inflammatory cell infiltration and reduction of edema after TanⅡA intervention (Figure 7C). Masson staining also showed a reduction in the area of fibrosis in the right ventricle of the hearts of both groups (Figure 7D). ECG results showed that TanⅡA effectively reduced T-wave amplitude of the mouse hearts in both groups (Figure S7A). These results suggest that TanⅡA could alleviate the structural and functional damage of the heart at high altitude caused by the abnormally high expression of ITGA2B.

Mechanistically, TanⅡA reduced the p65 level in both OE-HH and Ctrl-HH mice (Figure 7E), consistent with the reduction of inflammatory cytokine contents (Figure S7B). The levels of p-ILK and ITGA2B were also decreased after TanⅡA treatment (Figure 7F and G), which further supports the regulatory relationship between ITGA2B–ILK and NF-κB p65. Meanwhile, ELISA results showed that the levels of ITGA2B, CK-MB, and NT-proBNP in the peripheral blood of both OE-HH and Ctrl-HH mice were significantly decreased after TanⅡA treatment (Figure S7C), and the levels of lactic acid (Figure S7D) and MDA (Figure S7E) in the peripheral blood of both groups were also decreased. In contrast, the peripheral blood ATP levels increased after TanⅡA treatment (Figure S7F). These results suggest that TanⅡA alleviates the disturbance of cardiac energy supply at high altitude caused by abnormal ITGA2B expression.

To further clarify the molecular mechanism of TanⅡA, molecular docking analysis was performed to compare the binding ability of TanⅡA with ITGA2B, ILK, and p65. The results showed that TanⅡA binds ITGA2B most strongly with an affinity of −9.2 kcal/mol (Figure S7G). Then, we confirmed that TanⅡA can target ITGA2B by surface plasmon resonance (SPR), with a binding affinity of 44.9 μM (Figure S7H), which is considered moderate in strength for protein–small molecule interactions *in vitro*. Furthermore, molecular dynamics simulation (Figure S7I) and cellular thermal shift assay (CETSA) (Figure S7J) demonstrated the direct binding of TanⅡA to ITGA2B. Considering that the affinity (−9.2 kcal/mol) between TanⅡA and ITGA2B was close to that of ITGA2B with the positive drug tirofiban hydrochloride, we used tirofiban hydrochloride to further verify the role of ITGA2B in high-altitude myocardial injury. Echocardiogram results showed that specific inhibition of ITGA2B significantly increased the tricuspid annular plane systolic excursion in mice (Figure S7K). Tirofiban hydrochloride could significantly increase the ejection fraction and fractional shortening, and reduce the thickness of the right ventricular free wall (RVFW) of the heart in the Ctrl-HH mice (Figure S7L). H&E staining also showed that tirofiban hydrochloride alleviated hypobaric hypoxia-induced cardiac hypertrophy and reduced myocardial cell edema and vacuolar lesions in the Ctrl-HH mice (Figure S7M).

## Discussion

It is well known that native lowlanders have different adaptability to the high-altitude environment. The heart is an oxygen-intensive organ responsible for transporting blood, oxygen, and nutrients to various tissues throughout the body, and is highly susceptible to an oxygen-deficient condition. Prolonged hypoxia can lead to cardiac hypertrophy at high altitude. At the same time, cardiac hypoxia and ischemic injury will then easily result in pathological remodeling, leading to myocardial cell loss and fibrosis, which ultimately brings about cardiac function impairment. Thanks to the development of sequencing technology, plateau adaptation genes have been gradually discovered [4]. However, relatively few studies have been conducted on genes related to cardiac function maintenance at plateau, and the underlying molecular mechanisms of myocardial injury have not been elucidated.

Here, we established a cohort of 134 lowlanders and conducted DIA proteomic profiling of the plasma. A group of significantly upregulated proteins were identified in the H_A group, in which ITGA2B and ILK participate in a variety of biological processes and have an obvious distinguishing effect on the H_A and H_N populations. Functionally, we found that aberrant upregulation of ITGA2B resulted in metabolism disorders, increased secretion of inflammatory cytokines, and dysregulation of cardiac structure and function under hypoxic conditions. Mechanistically, ITGA2B promoted the phosphorylation of ILK and IκBα, leading to the upregulation of p65, which further accumulated in the nucleus and led to the production of inflammatory cytokines. Ultimately, ITGA2B increased inflammatory cell infiltration in the heart tissue, aggravated heart load, and caused excessive damage (Figure 7H).

Notably, ITGA2B and its downstream protein ILK showed significantly higher expression in the plasma of individuals with high-altitude myocardial abnormalities. Hypoxia increases the content of ILK and promotes its kinase activity to induce angiogenesis [29]. ILK can also enhance the transcriptional activity of NF-κB in cardiomyocytes and cause the secretion of inflammatory factors [11]. Meanwhile, circulating ITGA2B has been reported to have a connection with cardiac injury and prognosis, but this has not been experimentally confirmed and the underlying mechanisms remain unexplored [20–23]. Therefore, the ITGA2B–ILK axis may play an important role in hypoxic myocardial injury at high altitude. We overexpressed ITGA2B in the heart and found that ITGA2B OE indeed aggravated the structural and functional damage of the heart through the NF-κB signaling pathway and the glycolytic process under chronic hypoxic conditions. However, we did not have sufficient evidence to show that the highly expressed ITGA2B in the H_A group was derived from the chronically hypoxic cardiac tissue. Hypoxia can cause excessive platelet activation [30,31], resulting in the release of α-granules which contain the fibrinogen receptor αⅡbβ3, the collagen receptor GPVI, and the components of the von Willebrand factor (VWF) receptor complex GPIb-IX-V [32,33]. Further studies are needed to explore the source of ITGA2B accumulation in the heart under chronic hypoxia. In addition, gender may also affect the adaptation to the plateau environment. However, as our cohort included only male participants, future clinical work on female individuals is needed to determine whether the findings of this study can be generalized to women.

Tirofiban is an intravenously administered ITGA2B antagonist with a relatively short half-life which can reversibly bind to the arginine-glycine-aspartic acid (RGD) recognition site of ITGA2B, and specifically inhibit fibrinogen-dependent platelet aggregation [34]. A previous study has reported that tirofiban can reduce the incidence of thrombotic cardiovascular events [35], supporting its use in patients with unstable angina pectoris and non-Q wave myocardial infarction. In this study, we found that tirofiban also showed a good protective effect on myocardial abnormalities at high altitude. However, due to the strong antithrombotic effect of tirofiban, long-term use of tirofiban can easily cause abnormal coagulation function, and result in thrombocytopenia [36]. Therefore, we focused on the safer and more widely used traditional Chinese medicine. TanⅡA is the major lipid-soluble bioactive component in *Salvia miltiorrhiza* Bunge, and it has strong effects on inhibiting inflammatory response, anti-platelet aggregation, anti-oxidative stress, alleviating myocardial ischemia-reperfusion injury, and protecting cardiovascular function [28]. TanⅡA plays an important role in cardiovascular protection through mechanisms, such as regulating lipid metabolism [37], improving myocardial hypertrophy, reducing myocardial cell apoptosis, and inhibiting myocardial fibrosis [38]. Our results revealed that TanⅡA ameliorated the myocardial injury caused by ITGA2B OE and hypobaric hypoxia exposure in mice and modulated the activation of NF-κB via the ITGA2B–ILK signaling axis (Figure 7H).

## Conclusion

Although the function of ITGA2B in platelets has been extensively studied, the role of circulating ITGA2B in cardiac regulation remains a mystery. Our study uncovers a previously unknown role of ITGA2B in inducing cardiac inflammation by enhancing the transcriptional activity of NF-κB in heart tissues, which aggravates chronic hypoxia-induced cardiac overload. Furthermore, we demonstrate that TanⅡA could alleviate cardiac structural and functional damage caused by ITGA2B OE under hypobaric hypoxia. Our findings not only shed light on the impaired effects and underlying mechanisms of ITGA2B on the heart under hypoxic conditions, but also provide a theoretical basis for TanⅡA as a new candidate drug to counteract hypoxia-induced myocardial abnormalities.

## Materials and methods

### Sample collection

All participants were native lowlanders born and grew up below 2000 m altitude before 21 years old. They had not traveled to plains within the three months before the investigation. Detailed information is shown in Figure S1 and Table S1. Blood samples were collected from participants using blood collection tubes containing heparin sodium anticoagulant and then centrifuged at 3000 *g* for 5 min. The plasma was aspirated and stored at −80°C.

### Clinical data collection

The native lowlanders who had migrated to a 4500-m plateau were recruited, and blood routine examination, blood biochemistry test, echocardiography, and ECG were performed. Meanwhile, a questionnaire survey was conducted on residence duration in Xizang Autonomous Region, China, smoking status, alcohol consumption, sleep quality, and so on. Participants with BMI > 25 or < 18 kg/m^2^ were excluded. Participants were also required to be free from colds, fever, or medication use for two weeks prior to the study. Then, according to the geographical origin and dietary habits of the participants, healthy volunteers from Chengdu, Sichuan Province, China with similar living habits were selected as the control cohort. The plain control individuals had not traveled to high-altitude regions (> 2000 m) within the three months before the investigation. According to the distribution characteristics of BMI and age, a total of 134 subjects were enrolled including the plain control individuals.

### Proteome sample preparation

Plasma samples were prepared according to the methods previously described with some modifications [39]. Briefly, 2 μl of plasma sample from each participant was diluted in 20 μl of buffer containing 0.1 M NH_4_HCO_3_ and 6 M urea (Catalog No. U5378, Sigma-Aldrich, Saint Louis, MO) at 32°C for 30 min. Disulfide reduction of proteins was performed with 5 mM dithiothreitol (DTT) for 30 min at 32°C. Alkylation was carried out using 10 mM iodoacetamide (IAA) in the darkness for 45 min. The proteins were then diluted with 100 μl of 50 mM NH_4_HCO_3_, and digested with trypsin at 37°C overnight. The peptide mixtures were then acidified by 10% trifluoroacetic acid (TFA) and cleaned-up using SOLAμ (Catalog No. 62209-001, Thermo Fisher Scientific, Waltham, MA).

For AC16 cell samples, the proteins were extracted by sonication and dissolved in lysis buffer (8 M urea, 50 mM Tris-HCl pH 8.0, 50 mM NH_4_HCO_3_, 50 mM IAA, and 1 mM phenylmethanesulfonyl fluoride). The cell debris was removed by centrifugation, and proteins in the supernatant were collected and digested for proteomic analysis using the filter-aided sample preparation (FASP) method [40]. Briefly, 50 μg proteins were diluted with 400 μl of buffer containing 0.1 M NH_4_HCO_3_ and 6 M urea. Disulfide reduction was performed as described above. Then the proteins were digested with trypsin at 37°C overnight. The peptide mixtures were collected by centrifugation at room temperature, and then acidified with 10% TFA. Finally, the peptides were dissolved in loading buffer for liquid chromatography-tandem mass spectrometry (LC-MS/MS) analysis.

### LC-MS/MS analysis and database searching

Plasma peptides were analyzed by LC-MS/MS with the EASY-nLC 1200 system (Thermo Fisher Scientific, Bremen, Germany) coupled to a Q Exactive Quadrupole-Orbitrap Mass Spectrometer (Thermo Fisher Scientific, Bremen, Germany) in DIA mode. For each DIA acquisition, peptide mixtures were loaded onto a precolumn (3 μm, 20 mm × 75 μm) at a maximum pressure of 500 bar, followed by separation on a homemade analytical column (1.9 μm C18 reverse-phase particles, 30 cm × 75 μm) with a 120-min linear gradient. The *m/z* range of MS1 was 400–1200 with a resolution of 120,000, automatic gain control (AGC) target of 3E6, and maximum ion injection time of 80 ms. For DIA MS/MS scan, 32 sequential isolation windows were set up with the window size of 25 *m/z* to cover the survey scan range. For MS/MS, the resolution was set to 30,000. The raw files were analyzed using Spectronaut software (v15.7) in direct-DIA mode against the Swiss-Prot reviewed human library (2022-03 release). The analysis was conducted using the default settings, with the identification thresholds set at false discovery rate (FDR) < 1% at both peptide and protein levels. Detailed information about identified proteins in DIA-based proteomic data of 134 plasma samples is shown in Table S4.

Peptide mixtures from AC16 cells were similarly analyzed by an Orbitrap MS (Thermo Fisher Scientific, Bremen, Germany). The analytical gradient of the LC system was 120 min, and the MS/MS spectra were acquired using data-dependent acquisition (DDA) mode. The raw files were processed with the MaxQuant software (v2.0.3.0) [41], and the spectra were searched by the Andromeda search engine [42] against the Swiss-Prot reviewed human database. Full cleavage by trypsin was set including two mis-cleavages. The identification thresholds of peptides and proteins were set at FDR < 1%. The peak area was used to quantify peptide abundance with MaxQuant. For proteins, the “Peptides to Use” parameter was set to “Unique + Razor”. Protein quantitation information was calculated by the intensity of the top 3 peptides. Default settings were performed for other parameters. Detailed information of identified proteins in proteomic datasets of AC16 cells is shown in Table S5.

We employed the PRM method to perform targeted validation of key DEPs in plasma samples from 32 individuals belonging to an independent cohort. Similarly, a LC gradient of 120 min was adopted. According to DIA results, the peptides with higher abundance in DEPs were screened. The MS parameters mainly included retention time and the molecular weight of monitored parent ions.

### Proteomic data analysis

The protein quantitative information of 134 (30 P_N, 73 H_N, and 31 H_A) plasma samples was extracted from direct-DIA searching results to represent the expression level of proteins across samples. Before normalization, proteins with more than 75% missing values in all samples were removed, and missing values were imputed with the minimum value in each sample. Then, the expression matrix was subjected to variance stabilization normalization (VSN) via the vsn package (v3.62.0) [43] in R/Bioconductor, a method demonstrating robust performance for label-free quantitative proteomics [44]. The differential expression analysis for proteins was performed using the *limma* package (v3.50.3) [45] in R/Bioconductor. Proteins with |log_2_ fold change| > 1 and Benjamini–Hochberg adjusted *P* value < 0.05 were identified as DEPs. Functional enrichment of DEPs was assessed by clusterProfiler (v4.2.1) [46] for Gene Ontology (GO) biological processes and Kyoto Encyclopedia of Genes and Genomes (KEGG) pathways. The significance of enrichment for each term was determined by the Benjamini–Hochberg adjusted *P* value.

### ITGA2B OE in AC16 cells and C57BL/6 mice

AC16 human cardiomyocyte cell line was purchased from Xiamen Immocell Biotechnology Co., Ltd., China (Catalog No. IM-H478) and validated by the short tandem repeat (STR) profiling method. Cells were cultured in Dulbecco’s Modified Eagle Medium/Nutrient Mixture F-12 (DMEM-F12; Catalog No. 11320033, Thermo Fisher Scientific, Waltham, MA) supplemented with 10% (v/v) fetal bovine serum (FBS; Catalog No. A5256701, Thermo Fisher Scientific, Waltham, MA) and 1% (v/v) penicillin/streptomycin (Catalog No. 15140122, Thermo Fisher Scientific, Waltham, MA), and maintained in a humidified atmosphere at 37°C with 5% CO_2_ under either normoxic (21% O_2_, N) or hypoxic (1% O_2_, H) conditions, using the Baker Ruskinn InvivO_2_ hypoxia workstations. 293T cells were transfected with the OE plasmid pCDH-CMV-ITGA2B-3×FLAG-CopGFP-Puro (Catalog No. YBK20230425, Yike Biotechnology, Beijing, China) and two helper plasmids using lipo3000 transfection reagent (Catalog No. L3000015, Thermo Fisher Scientific, Waltham, MA). The GFP fluorescence intensity of 293T cells was observed, and the cell culture medium was collected. According to GFP fluorescence intensity, 48-h and 72-h cell culture media were pooled and concentrated to enrich the packaged virus particles. Then the concentrated virus particles were used to infect AC16 cells, and the cell line stably overexpressing ITGA2B was screened by 4 μg/ml puromycin (Catalog No. A1113803, Thermo Fisher Scientific, Waltham, MA) for 3–5 passages.

Eight-week-old male C57BL/6 mice were purchased from Beijing Vital River Laboratory Animal Technology Co., Ltd., China. Mice were divided into six groups: AAV9-FLAG under normoxic conditions (Ctrl), AAV9-ITGA2B-FLAG under normoxic conditions (OE), AAV9-FLAG with hypobaric hypoxia exposure (Ctrl-HH), AAV9-ITGA2B-FLAG with hypobaric hypoxia exposure (OE-HH), AAV9-FLAG with hypobaric hypoxia exposure + TanⅡA treatment (Ctrl-HH-TanⅡA), and AAV9-ITGA2B-FLAG with hypobaric hypoxia exposure + TanⅡA treatment (OE-HH-TanⅡA). AAV9 (*CTNI*) carrying the *ITGA2B* sequence or empty AAV9 was injected (8–10 μl/site, 1 × 10^12^ viral particles/mouse) into mouse cardiac apex by thoracic operation one month before hypobaric hypoxia exposure. Except for the Ctrl and OE groups, the other four groups were subjected to a hypobaric hypoxia simulation chamber (ProOx-810, Tow-Int Tech, Shanghai, China) for 40 consecutive days at simulated altitude of 6000 m, cabin pressure of 47.5 kPa, oxygen partial pressure of 9.6 kPa at 25°C. The mice were given saline or TanⅡA by gavage daily.

### Detection of mouse echocardiography and ECG

After 40-day hypobaric hypoxia exposure, mice were anesthetized with 3%–5% isoflurane using an animal anesthesia machine (VMR, Beijing Yeeran Technology, Beijing, China). The mice were then fixed supine on the experimental table, and their chest hair was removed using an electric shaver and depilation cream, with the shaved area extending from the neck to the manubrium sternum and bilaterally to the axillary midline. After hair removal, the coupling solvent was applied to the chest skin, and echocardiographic data were collected when the mice exhibited a stable heart rate at 400–500 beats/min and body temperature at 35°C–37°C. The long axis section of the left ventricle of the mice was taken, and the M-ultrasound was performed. Left ventricular ejection fraction, left ventricular fractional shortening, LVPWd, and IVSd were measured. Then ECG was measured by a multi-channel physiological signal acquisition and processing system. The temperature of the operating table on which the mice were placed was maintained at 37°C throughout the assay. After the aforementioned experiments, the mice were sacrificed and key blood routine indicators of each mouse are recorded in Table S6.

### Observation of intracellular ultrastructure

The cells were fixed with fixing solution at 4°C and pelleted by centrifugation. After washing three times (15 min per wash) with phosphate buffered saline (PBS), the cell samples were dehydrated through a graded series of ethanol and acetone. The cell samples were then embedded using a mixture of acetone and embedding agent (Catalog No. 90529-77-4, SPI Pharma, Wilmington, DE). Subsequently, the samples were prepared into 60–80 nm ultrathin sections using an ultramicrotome (UC7, Leica, Wetzlar, Germany), and the sections were stained with 2% uranium acetate followed by lead citrate. Finally, the ultrastructural changes in the AC16 cells subjected to hypoxia and ITGA2B OE were observed using the HT7700 Transmission Electron Microscope (HITACHI, Tokyo, Japan), and the images were collected for analysis.

### Glucose uptake analysis

Ctrl and OE AC16 cells were seeded into 6-well plates at a density of 3 × 10^5^ cells per well. Cells from both groups were cultured under three conditions: 37°C, 5% CO_2_, 1% O_2_ for 0 h; 37°C, 5% CO_2_, 1% O_2_ for 24 h; and 37°C, 5% CO_2_, 1% O_2_ for 48 h. Following culture, the cells were washed twice with preheated glucose-free DMEM/F12 medium, and then incubated in preheated glucose-free medium for 15 min at 37°C in an incubator with 5% CO_2_ and 21% O_2_. The glucose uptake capacity was measured using a glucose uptake assay kit (Catalog No. UP03, DOJINDO, Kyushu Island, Japan). In brief, following medium removal, the cells were incubated with preheated Probe Solution. After three washes with precooled WI Solution, glucose uptake was quantified by measuring red fluorescence intensity using fluorescence microscopy.

### Seahorse assay

Glycolytic stress test was performed using the Seahorse XF96 Extracellular Flux Analyzer (Agilent, Santa Clara, CA) according to the manufacturer’s instructions. Briefly, AC16 cells (6000 cells/well) were seeded in 96-well XF cell culture microplates containing DMEM/F12 medium. The microplates were incubated for 24 h under normoxic (37°C, 5% CO_2_, 21% O_2_) or hypoxic (37°C, 5% CO_2_, 1% O_2_) conditions. Extracellular acidification rate (ECAR) was measured in XF base medium (pH 7.4) containing 10 mM glucose, 2 mM glutamine, 1 mM sodium pyruvate, and 1 mM N-2-hydroxyethylpiperazine-N-2-ethane sulfonic acid (HEPES) buffer following sequential additions of oligomycin (1.5 μM) and 2-deoxy-glucose (2-DG: 50 mM). Data were analyzed using the Seahorse XF Glycolysis Stress Test Report Generator package.

### RT-qPCR

Total RNA was extracted from AC16 cells and mouse heart tissues of different groups for *ITGA2B* expression validation. RNA quality and quantity were assessed using agarose gel electrophoresis. For each sample, about 500 ng of RNA was reversely transcribed to complementary DNA (cDNA) using the High-Capacity cDNA Reverse Transcription Kit (Catalog No. 4368814, Thermo Fisher Scientific, Waltham, MA). RT-qPCR was performed using the SYBR Green Master Mix (Catalog No. 11200ES08, Yeasen Biotechnology, Shanghai, China). Relative mRNA expression levels were calculated by the 2^−ΔΔCt^ method.

### WB assay

AC16 cells and mouse heart tissues were lysed using cold Radio Immunoprecipitation Assay (RIPA) buffer supplemented with protease inhibitors (Catalog No. HY-K0013, MCE, Monmouth Junction, NJ) for protein extraction. Equal amounts of protein (50 µg per sample) were mixed with 5× sodium dodecyl sulfate-polyacrylamide gel electrophoresis (SDS-PAGE) loading buffer and denatured at 95°C for 5 min. Proteins were separated on 10% SDS-PAGE and transferred onto polyvinylidene fluoride (PVDF) membranes. Appropriate primary antibodies were incubated with the PVDF membranes overnight at 4°C, including anti-ITGA2B (Catalog No. ab134131, Abcam, Cambridge, UK), anti-FLAG (Catalog No. M185-3, MBL, Beijing, China), anti-β-Tubulin (Catalog No. 2146S, Cell Signaling Technology, Danvers, MA), anti-β-Actin (Catalog No. 66009-1-Ig, Proteintech, Wuhan, China), anti-ILK (Catalog No. 12955-1-AP, Proteintech), anti-IκBα (Catalog No. 4814, Cell Signaling Technology), anti-p65 (Catalog No. 8242, Cell Signaling Technology), anti-p-ILK (Catalog No. AB1076, Merck, Darmstadt, Germany), and anti-p-IκBα (Catalog No. 2859, Cell Signaling Technology). On the following day, the PVDF membranes were washed with Tris-buffered saline with Tween 20 (TBST) buffer, and incubated with the secondary antibodies (Catalog No. SA00001-2 and SA00001-1, Proteintech). Finally, the protein bands were visualized by an exposure apparatus. For immunofluorescence experiments, the same antibodies were used as those for WB described above.

### Molecular docking

We retrieved the three-dimensional (3D) structures of ITGA2B, ILK, and p65 from the Research Collaboratory for Structural Bioinformatics (RCSB) Protein Data Bank (PDB). Using PyMOL (v1.8) and AutoDockTools (v1.5.6), we proceeded with modifications to remove native ligands and water molecules, add hydrogen atoms, and patch amino acids. The mol2 structure of the TanⅡA was made rotatable and converted to “PDBQT” format using AutoDockTools. Molecular docking was then executed using AutoDock-Vina software (v1.1.2). We visualized the docking results with PyMOL (v1.8) and analyzed the docking interaction patterns.

### SPR

The SPR experiment was performed on a Biacore 1K system (Cytiva, Uppsala, Sweden). The brief steps were as follows. After the solid-phase chip activation, the ITGA2B protein (Catalog No. HY-P77716, MCE, Monmouth Junction, NJ) was fixed to the chip at a flow rate of 5 μl/min for 420 s. Then, ethanolamine (Catalog No. 35063, Cytiva) was injected to seal at a rate of 10 μl/min. TanⅡA was diluted in gradient and the injection parameters were set as follows: 60 s contact, 120 s dissociation, and 30 s regeneration, with a flow rate of 30 μl/min at 25°C. Biacore Insight Evaluation (v5.0.18.22102) was used for analysis.

### Statistical analysis

Statistical comparisons between two groups and more than two groups were conducted using Student’s *t*-test and one-way analysis of variance (ANOVA) followed by Tukey’s post hot test, respectively, using GraphPad Prism (v8.3.0; GraphPad Software, La Jolla, CA). Data are presented as mean ± standard deviation (SD).

## Ethical statement

Plasma samples were collected following written informed consent obtained from all participants, and all related protocols were approved by the Ethics Committee of the Beijing Institute of Radiation Medicine, China (Approval No. AF/SC-08/02.153). Animal experiments were conducted in compliance with national and institutional guidelines and were approved by the Animal Ethics Committee of the Academy of Military Medical Sciences, China (Approval No. IACUC-DWZX-2022-610).

## Supplementary Material

qzaf030_Supplementary_Data

## Data Availability

DIA proteomic data of plasma samples and AC16 cells have been deposited to the ProteomeXchange Consortium [47] via the iProX [48] partner repository (ProteomeXchange: PXD047926 for plasma samples and PXD048442 for AC16 cells). The PRM data of the independent cohort have also been submitted to the ProteomeXchange Consortium (ProteomeXchange: PXD057518).

## CRediT author statement

**Yihao Wang:** Formal analysis, Funding acquisition, Writing – original draft, Writing – review & editing, Validation. **Pan Shen:** Data curation, Formal analysis, Visualization, Validation, Writing – review & editing. **Zhenhui Wu:** Formal analysis, Methodology, Investigation, Writing – review & editing, Validation. **Bodan Tu:** Methodology, Investigation, Validation. **Cheng Zhang:** Investigation. **Yongqiang Zhou:** Investigation. **Yisi Liu:** Methodology. **Guibin Wang:** Software. **Zhijie Bai:** Methodology. **Xianglin Tang:** Methodology. **Chengcai Lai:** Methodology. **Haitao Lu:** Writing – review & editing, Methodology. **Wei Zhou:** Investigation, Funding acquisition, Project administration, Resources, Supervision, Writing – review & editing. **Yue Gao:** Funding acquisition, Conceptualization, Project administration, Resources, Supervision, Writing – review & editing. All authors have read and approved the final manuscript.

## References

[1] Frisancho AR. Developmental functional adaptation to high altitude: review. Am J Hum Biol 2013;25:151–68. 24065360 10.1002/ajhb.22367

[2] Mallet RT , Burtscher J, Pialoux V, Pasha Q, Ahmad Y, Millet GP, et al Molecular mechanisms of high-altitude acclimatization. Int J Mol Sci 2023;24:1698. 36675214 10.3390/ijms24021698 PMC9866500

[3] Sharma V , Varshney R, Sethy NK. Human adaptation to high altitude: a review of convergence between genomic and proteomic signatures. Hum Genomics 2022;16:21. 35841113 10.1186/s40246-022-00395-y PMC9287971

[4] Storz JF , Cheviron ZA. Physiological genomics of adaptation to high-altitude hypoxia. Annu Rev Anim Biosci 2021;9:149–71. 33228375 10.1146/annurev-animal-072820-102736 PMC8287974

[5] Villafuerte FC , Simonson TS, Bermudez D, León-Velarde F. High-altitude erythrocytosis: mechanisms of adaptive and maladaptive responses. Physiology 2022;37:175–86. 10.1152/physiol.00029.2021 PMC9191173 35001654

[6] Richalet JP , Hermand E, Lhuissier FJ. Cardiovascular physiology and pathophysiology at high altitude. Nat Rev Cardiol 2024;21:75–8. 37783743 10.1038/s41569-023-00924-9

[7] Penaloza D , Arias-Stella J. The heart and pulmonary circulation at high altitudes. Circulation 2007;115:1132–46. 17339571 10.1161/CIRCULATIONAHA.106.624544

[8] Virani SS , Alonso A, Benjamin EJ, Bittencourt MS, Callaway CW, Carson AP, et al Heart disease and stroke statistics — 2020 update: a report from the American Heart Association. Circulation 2020;141:e139–596. 31992061 10.1161/CIR.0000000000000757

[9] Bae J , Salamon RJ, Brandt EB, Paltzer WG, Zhang Z, Britt EC, et al Malonate promotes adult cardiomyocyte proliferation and heart regeneration. Circulation 2021;143:1973–86. 33666092 10.1161/CIRCULATIONAHA.120.049952 PMC8131241

[10] Cooper J , Giancotti FG. Integrin signaling in cancer: mechanotransduction, stemness, epithelial plasticity, and therapeutic resistance. Cancer Cell 2019;35:347–67. 30889378 10.1016/j.ccell.2019.01.007 PMC6684107

[11] Yue Y , Wang C, Benedict C, Huang G, Truongcao M, Roy R, et al Interleukin-10 deficiency alters endothelial progenitor cell-derived exosome reparative effect on myocardial repair via integrin-linked kinase enrichment. Circ Res 2020;126:315–29. 31815595 10.1161/CIRCRESAHA.119.315829 PMC7015105

[12] Pulikkot S , Hu L, Chen Y, Sun H, Fan Z. Integrin regulators in neutrophils. Cells 2022;11:2025. 35805108 10.3390/cells11132025 PMC9266208

[13] Marchini T , Mitre LS, Wolf D. Inflammatory cell recruitment in cardiovascular disease. Front Cell Dev Biol 2021;9:635527. 33681219 10.3389/fcell.2021.635527 PMC7930487

[14] Zhang X , Dong Y, Zhao M, Ding L, Yang X, Jing Y, et al ITGB2-mediated metabolic switch in CAFs promotes OSCC proliferation by oxidation of NADH in mitochondrial oxidative phosphorylation system. Theranostics 2020;10:12044–59. 33204328 10.7150/thno.47901 PMC7667693

[15] Ata R , Antonescu CN. Integrins and cell metabolism: an intimate relationship impacting cancer. Int J Mol Sci 2017;18:189. 28106780 10.3390/ijms18010189 PMC5297821

[16] Harston RK , Kuppuswamy D. Integrins are the necessary links to hypertrophic growth in cardiomyocytes. J Signal Transduct 2011;2011:521742. 21637377 10.1155/2011/521742 PMC3101892

[17] van den Kerkhof DL , van der Meijden PEJ, Hackeng TM, Dijkgraaf I. Exogenous integrin αⅡbβ3 inhibitors revisited: past, present and future applications. Int J Mol Sci 2021;22:3366. 33806083 10.3390/ijms22073366 PMC8036306

[18] Linscheid N , Poulsen PC, Pedersen ID, Gregers E, Svendsen JH, Olesen MS, et al Quantitative proteomics of human heart samples collected *in vivo* reveal the remodeled protein landscape of dilated left atrium without atrial fibrillation. Mol Cell Proteomics 2020;19:1132–44. 32291283 10.1074/mcp.RA119.001878 PMC7338087

[19] Doll S , Dreßen M, Geyer PE, Itzhak DN, Braun C, Doppler SA, et al Region and cell-type resolved quantitative proteomic map of the human heart. Nat Commun 2017;8:1469. 29133944 10.1038/s41467-017-01747-2 PMC5684139

[20] Tang S , Liu Y, Liu B. Integrated bioinformatics analysis reveals marker genes and immune infiltration for pulmonary arterial hypertension. Sci Rep 2022;12:10154. 35710932 10.1038/s41598-022-14307-6 PMC9203517

[21] Qian C , Chang D, Li H, Wang Y. Identification of potentially critical genes in the development of heart failure after ST-segment elevation myocardial infarction (STEMI). J Cell Biochem 2019;120:7771–7. 30485493 10.1002/jcb.28051

[22] Wingrove JA , Fitch K, Rhees B, Rosenberg S, Voora D. Peripheral blood gene expression signatures which reflect smoking and aspirin exposure are associated with cardiovascular events. BMC Med Genomics 2018;11:1. 29329538 10.1186/s12920-017-0318-6 PMC5767057

[23] Lapić I , Radić Antolic M, Horvat I, Premužić V, Palić J, Rogić D, et al Association of polymorphisms in genes encoding prothrombotic and cardiovascular risk factors with disease severity in COVID-19 patients: a pilot study. J Med Virol 2022;94:3669–75. 35415903 10.1002/jmv.27774 PMC9088581

[24] Palstrøm NB , Matthiesen R, Rasmussen LM, Beck HC. Recent developments in clinical plasma proteomics — applied to cardiovascular research. Biomedicines 2022;10:162. 35052841 10.3390/biomedicines10010162 PMC8773619

[25] He S , Zhang Q, Wu F, Chen J, He S, Ji Z, et al Influence of cigarettes on myocardial injury in healthy population after exposure to high altitude over 5000 m. Sci Total Environ 2023;855:158824. 36122711 10.1016/j.scitotenv.2022.158824

[26] Yang J , Jia Z, Song X, Shi J, Wang X, Zhao X, et al Proteomic and clinical biomarkers for acute mountain sickness in a longitudinal cohort. Commun Biol 2022;5:548. 35668171 10.1038/s42003-022-03514-6 PMC9170681

[27] Gao Z , Luo G, Ni B. Progress in mass spectrometry-based proteomics in hypoxia-related diseases and high-altitude medicine. OMICS 2017;21:305–13. 28486083 10.1089/omi.2016.0187

[28] Guo R , Li L, Su J, Li S, Duncan SE, Liu Z, et al Pharmacological activity and mechanism of tanshinone ⅡA in related diseases. Drug Des Devel Ther 2020;14:4735–48. 10.2147/DDDT.S266911 PMC7653026 33192051

[29] Lee SP , Youn SW, Cho HJ, Li L, Kim TY, Yook HS, et al Integrin-linked kinase, a hypoxia-responsive molecule, controls postnatal vasculogenesis by recruitment of endothelial progenitor cells to ischemic tissue. Circulation 2006;114:150–9. 16818815 10.1161/CIRCULATIONAHA.105.595918

[30] Delaney C , Davizon-Castillo P, Allawzi A, Posey J, Gandjeva A, Neeves K, et al Platelet activation contributes to hypoxia-induced inflammation. Am J Physiol Lung Cell Mol Physiol 2021;320:L413–21. 33264579 10.1152/ajplung.00519.2020 PMC8294621

[31] Zhang W , Ren H, Xu C, Zhu C, Wu H, Liu D, et al Hypoxic mitophagy regulates mitochondrial quality and platelet activation and determines severity of I/R heart injury. Elife 2016;5:e21407. 27995894 10.7554/eLife.21407 PMC5214169

[32] Berger G , Massé JM, Cramer EM. Alpha-granule membrane mirrors the platelet plasma membrane and contains the glycoproteins Ib, IX, and V. Blood 1996;87:1385–95. 8608228

[33] Suzuki H , Murasaki K, Kodama K, Takayama H. Intracellular localization of glycoprotein VI in human platelets and its surface expression upon activation. Br J Haematol 2003;121:904–12. 12786802 10.1046/j.1365-2141.2003.04373.x

[34] RESCUE BT Trial Investigators, Qiu Z, Li F, Sang H, Luo W, Liu S, et al Effect of intravenous tirofiban *vs*. placebo before endovascular thrombectomy on functional outcomes in large vessel occlusion stroke: the RESCUE BT randomized clinical trial. JAMA 2022;328:543–53. 35943471 10.1001/jama.2022.12584 PMC9364124

[35] King S , Short M, Harmon C. Glycoprotein Ⅱb/Ⅲa inhibitors: the resurgence of tirofiban. Vascul Pharmacol 2016;78:10–6. 26187354 10.1016/j.vph.2015.07.008

[36] Wang J , Zou D. Tirofiban-induced thrombocytopenia. Ann Med 2023;55:2233425. 37439782 10.1080/07853890.2023.2233425 PMC10348023

[37] Hu Q , Wei B, Wei L, Hua K, Yu X, Li H, et al Sodium tanshinone ⅡA sulfonate ameliorates ischemia-induced myocardial inflammation and lipid accumulation in Beagle dogs through NLRP3 inflammasome. Int J Cardiol 2015;196:183–92. 26143630 10.1016/j.ijcard.2015.05.152

[38] Huang X , Deng H, Shen QK, Quan ZS. Tanshinone ⅡA: pharmacology, total synthesis, and progress in structure-modifications. Curr Med Chem 2022;29:1959–89. 34749607 10.2174/0929867328666211108110025

[39] Tu B , Wang Y, Wu Z, Zhou W, Tang X, Zhang C, et al DIA-based serum proteomics revealed the protective effect of modified siwu decoction against hypobaric hypoxia. J Ethnopharmacol 2024;319:117303. 37827297 10.1016/j.jep.2023.117303

[40] Wiśniewski JR , Zougman A, Nagaraj N, Mann M. Universal sample preparation method for proteome analysis. Nat Methods 2009;6:359–62. 19377485 10.1038/nmeth.1322

[41] Cox J , Mann M. MaxQuant enables high peptide identification rates, individualized p.p.b.-range mass accuracies and proteome-wide protein quantification. Nat Biotechnol 2008;26:1367–72. 19029910 10.1038/nbt.1511

[42] Cox J , Neuhauser N, Michalski A, Scheltema RA, Olsen JV, Mann M. Andromeda: a peptide search engine integrated into the MaxQuant environment. J Proteome Res 2011;10:1794–805. 21254760 10.1021/pr101065j

[43] Huber W , von Heydebreck A, Sültmann H, Poustka A, Vingron M. Variance stabilization applied to microarray data calibration and to the quantification of differential expression. Bioinformatics 2002;18:S96–104. 12169536 10.1093/bioinformatics/18.suppl_1.s96

[44] Välikangas T , Suomi T, Elo LL. A systematic evaluation of normalization methods in quantitative label-free proteomics. Brief Bioinform 2018;19:1–11. 27694351 10.1093/bib/bbw095 PMC5862339

[45] Ritchie ME , Phipson B, Wu D, Hu Y, Law CW, Shi W, et al limma powers differential expression analyses for RNA-sequencing and microarray studies. Nucleic Acids Res 2015;43:e47. 25605792 10.1093/nar/gkv007 PMC4402510

[46] Wu T , Hu E, Xu S, Chen M, Guo P, Dai Z, et al clusterProfiler 4.0: a universal enrichment tool for interpreting omics data. Innovation (Camb) 2021;2:100141. 34557778 10.1016/j.xinn.2021.100141 PMC8454663

[47] Vizcaíno JA , Deutsch EW, Wang R, Csordas A, Reisinger F, Ríos D, et al ProteomeXchange provides globally coordinated proteomics data submission and dissemination. Nat Biotechnol 2014;32:223–6. 24727771 10.1038/nbt.2839 PMC3986813

[48] Ma J , Chen T, Wu S, Yang C, Bai M, Shu K, et al iProX: an integrated proteome resource. Nucleic Acids Res 2019;47:D1211–7. 30252093 10.1093/nar/gky869 PMC6323926

