# Supplementary material for: Plasma Proteomic Profiling Reveals ITGA2B as A Key Regulator of Heart Health in High-altitude Settlers
Source: Genomics Proteomics Bioinformatics. 2025 Apr 8;23(2):qzaf030. doi: 10.1093/gpbjnl/qzaf030 (PMC12417084; doi:10.1093/gpbjnl/qzaf030)
Supplement: qzaf030_Supplementary_Data [file qzaf030_supplementary_data.zip › Supplementary material captions.rtf]

Supplementary material
Figure S1  Detailed clinical characteristics of the cohort and proteome data quality metric
A. The years of settlement of H_N and H_A individuals in the plateau. B. The blood oxygen saturation was significantly reduced in H_N and H_A. C. Partial results of peripheral blood routine test from P_N, H_N, and H_A. D. Content changes of blood lipid in peripheral blood. E.–G. Distribution of peptide number (E), P values (F) and C-score (G) for identified proteins in the proteome dataset. H. Pearson correlation analysis of three proteome datasets. SaO2, oxygen saturation in arterial blood; RBC, red blood cell; HCT, hematokrit; WBC, white blood cell; PLT, platelet; TC, total cholesterol;TG, triglyceride; HDL, high density lipoprotein; LDL, low density lipoprotein.

Figure S2  Functional differences of DEPs compared among different groups
A. The overall expression change in H_N vs. P_N, H_A vs. H_N and H_A vs. P_N. The red and blue line represented 1 and −1, respectively. B. The overlap of DEPs among different categories. C. Distribution of upregulated and downregulated DEPs among different categories. D. The enriched biological processes and KEGG pathways in each category. Grey, NA.

Figure S3  Relationship of myocardial abnormalities and residence time on the plateau
A. The change trend of CK-MB content in high-altitude settlers with the increasing time of plateau settlement. B. The number and proportion of people with abnormal CK-MB content increased with the residence time. C. UMAP analysis of people with different residence time on the plateau. D. and E. The overall content change (D) and the content change at different time periods (E) of 10 key DEPs in proteome data. F. Content validation of the other 8 key DEPs in plasma by ELISA. G. Content change of the other 8 key DEPs in plasma by ELISA at different time periods.

Figure S4  Construction of cell lines and mouse models overexpressing ITGA2B protein
A. Green fluorescence images showed that ITGA2B was overexpressed in the AC16 cells. B. The level of ITGA2B mRNA in AC16 cells and mouse hearts was determined by RT‐qPCR. C. Western blot analysis of ITGA2B in AC16 cells and mouse hearts. D. Proteomic analysis workflow for discovering DEPs in four groups of AC16 cells. E. Proteomic data indicated significantly increase of ITGA2B protein abundance in OE AC16 cells. F. ELISA results showed that ITGA2B was highly expressed in heart tissues after hypoxic exposure and AAV9 infection. G. and H. Content change of myocardial injury-related indicators in mice serum for CK-MB (G) and NT-proBNP (H). I. CK-MB content was increased in culture medium supernatant of OE-H AC16 cells. AAV9, adeno-associated virus serotype 9.  

Figure S5  Overexpression of ITGA2B aggravated hypoxic injury in AC16 cells
A. and B. ITGA2B OE combined with chronic hypoxia enlarged heart tissues (A) and increased cardiac organ coefficient in mice (B). C. Masson staining was used to detect fibrosis in the heart tissue of mice with chronic hypoxic exposure. D. Content change of myocardial injury-related indicators in culture medium supernatant of AC16 cells. E. Flow cytometry was used to determine the cell size and granularity of AC16 cells. F. Results of SSC/FSC showed that ITGA2B OE led to a significant increase in the granularity of AC16 cells after hypoxic exposure. G. PCA to detect proteomic differences of AC16 cells. PCA, principal component analysis; PC, principal component; SSC, side scatter; FSC, forward scatter; SSC-A, SSC-area; FSC-A, FSC-area; CTNI, cardiac troponin I.

Figure S6  OE of ITGA2B increased the production of inflammatory factors and oxidative stress
A. and B. The level of IL-6 mRNA was determined by RT‐qPCR in AC16 cells (A) and mouse heart (B) after prolonged hypoxia. C. and D. Content change of inflammatory factors in culture medium of AC16 cells (C) and mouse hearts (D). E. Representative transmission electron microscope micrographs of mitochondria in OE AC16 cells and Ctrl AC16 cells with (H) or without hypoxic exposure under magnification 1200× (upper panels) and 5000× (lower panels), respectively. F. OE of ITGA2B aggravated the hypoxia-induced increased in MDA content. G. Representative images of ROS production in AC16 cells with or without hypoxic exposure. H. OE of ITGA2B combined with hypoxia exposure caused a decrease in ATP amount in AC16 cells (left) and mouse hearts (right). TNF, tumor necrosis factor; ATP, adenosine triphosphate; MDA, malondialdehyde; ROS, reactive oxygen species.

Figure S7  TanⅡA relieved inflammation response and oxidative stress caused by abnormally high-expressed ITGA2B and chronic hypoxic exposure
A. ECG results showed that TanⅡA decreased the T-wave amplitude of mice. B. TanⅡA reduces the production of inflammatory factors in heart tissues. C. ELISA results showed that TanⅡA significantly decreased the levels of ITGA2B, CK-MB, and NT-proBNP in serum. D. and E. TanⅡA significantly reduced the content of Lac (D) and MDA (E) in heart tissue. F. The content of ATP in heart tissue was significantly increased after TanⅡA intervention. G. Molecular docking of TanⅡA with ITGA2B protein. H. TanⅡA specifically binds to ITGA2B in SPR assay. I. Molecular dynamics simulation of ITGA2B and TanⅡA. J. Cellular thermal shift assay revealed that TanⅡA delayed the degradation of ITGA2B. K. Representative ECG after intervention of Trio. L. Tiro significantly increased EF and FS and reduce RVFW thickness of mice with chronic hypoxic exposure. M. The hematoxylin–eosin staining was used to determine the pathological damage of cardiac tissue. SPR, surface plasmon resonance; Trio, tirofiban hydrochloride; RMSD, root mean square deviation; DMSO, dimethyl sulfoxide; GAPDH, glyceraldehyde-3-phosphate dehydrogenase; RVFW, right ventricular free wall; LVEF, left ventricular ejection fraction.
 
Table S1  Demographics and baseline characteristics of the study populations

Table S2  Clustering of 575 proteins based on the results of differentially expressed analysis

Table S3  The differentially expressed analysis results between the ITGA2B OE and Ctrl AC16 cells under both normal (N) and hypoxic (H) conditions 

Table S4  Detailed information about identified proteins in DIA-based proteomics of 134 plasma samples 

Table S5  Detailed information of identified proteins in proteomic datasets of AC16 cells

Table S6  Key blood routine indicators of each mouse in the 40-day chronic hypoxia exposure experiment
