# Supplementary material for: Plasma Proteomic Profiling Reveals ITGA2B as A Key Regulator of Heart Health in High-altitude Settlers
Source: Genomics Proteomics Bioinformatics. 2025 Apr 8;23(2):qzaf030. doi: 10.1093/gpbjnl/qzaf030 (PMC12417084; doi:10.1093/gpbjnl/qzaf030)
Supplement: qzaf030_Supplementary_Data [file qzaf030_supplementary_data.zip › Table S1.docx]

| **Table S1 Demographics and baseline characteristics of the study populations** | | | | |
| --- | --- | --- | --- | --- |
| **Variables** | **Plain cohort (500 m, *n* = 30)** | **Highland cohort (4500 m)** | | |
|  |  |  |  |  |
|  |  | **Total (*n* = 104)** | **Non-myocardial injury (*n* = 73)** | **Myocardial injury (*n* = 31)** |
|  |  |  |  |  |
| **Age (years)** |  |  |  |  |
| Mean ± SD | 23.2 ± 2.1 | 23.5 ± 2.1 | 23.4 ± 2.2 | 23.6 ± 2.2 |
| Median (IQR) | 23.0 (22.0–25.0) | 23.0 (22.0–25.0) | 23.0 (22.0–25.0) | 23.0 (22.0–25.0) |
| Range | 21.0–28.0 | 19.0–28.0 | 19.0–28.0 | 20.0–28.0 |
| **BMI** |  |  |  |  |
| Mean ± SD | 21.6 ± 1.7 | 21.5 ± 1.7 | 21.5 ± 1.7 | 21.5 ± 1.7 |
| Median (IQR) | 21.2 (20.4–23.0) | 21.1 (20.3–23.0) | 21.1 (20.3–23.0) | 21.1 (20.3–23.0) |
| Range | 18.6–24.9 | 18.6–25.0 | 18.6–25.0 | 18.7–24.7 |
| **Time spent on the plateau (years)** | | |  |  |
| Mean ± SD |  | 4.9 ± 2.8 | 4.9 ± 2.1 | 5.3 ± 2.1 |
| Median (IQR) |  | 4.0 (2.0–5.8) | 5.0 (3.0–6.0) | 5.0 (4.0–7.0) |
| Range |  | 1.0–11.0 | 1.0–10.0 | 2.0–11.0 |

*Note*: BMI, body mass index; SD, standard deviation; IQR, interquartile range.
