# Supplementary figures and images for: Plasma Proteomic Profiling Reveals ITGA2B as A Key Regulator of Heart Health in High-altitude Settlers
Source: Genomics Proteomics Bioinformatics. 2025 Apr 8;23(2):qzaf030. doi: 10.1093/gpbjnl/qzaf030 (PMC12417084; doi:10.1093/gpbjnl/qzaf030)

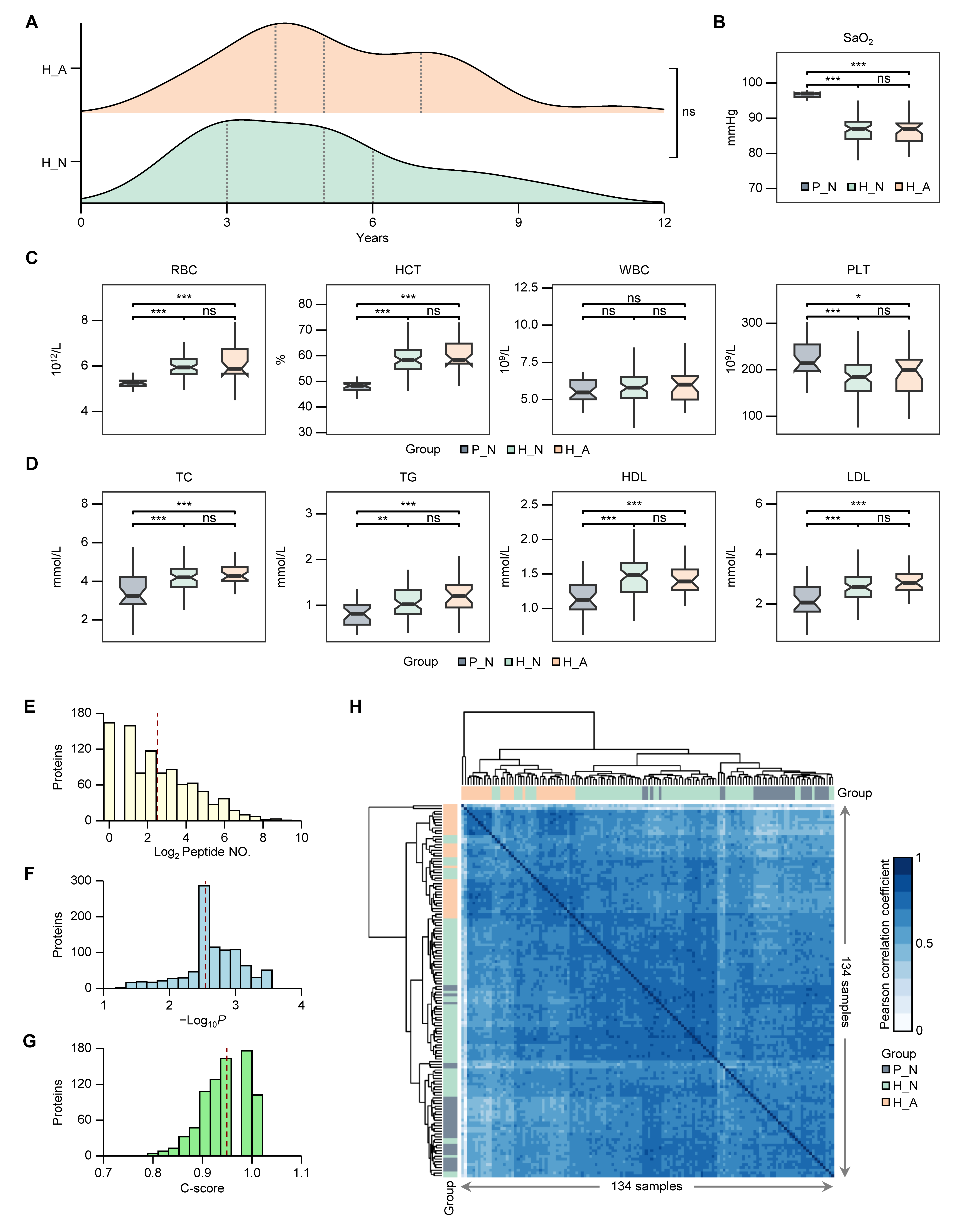

Supplement: qzaf030_Supplementary_Data [file qzaf030_supplementary_data.zip › Figure S1.tif]

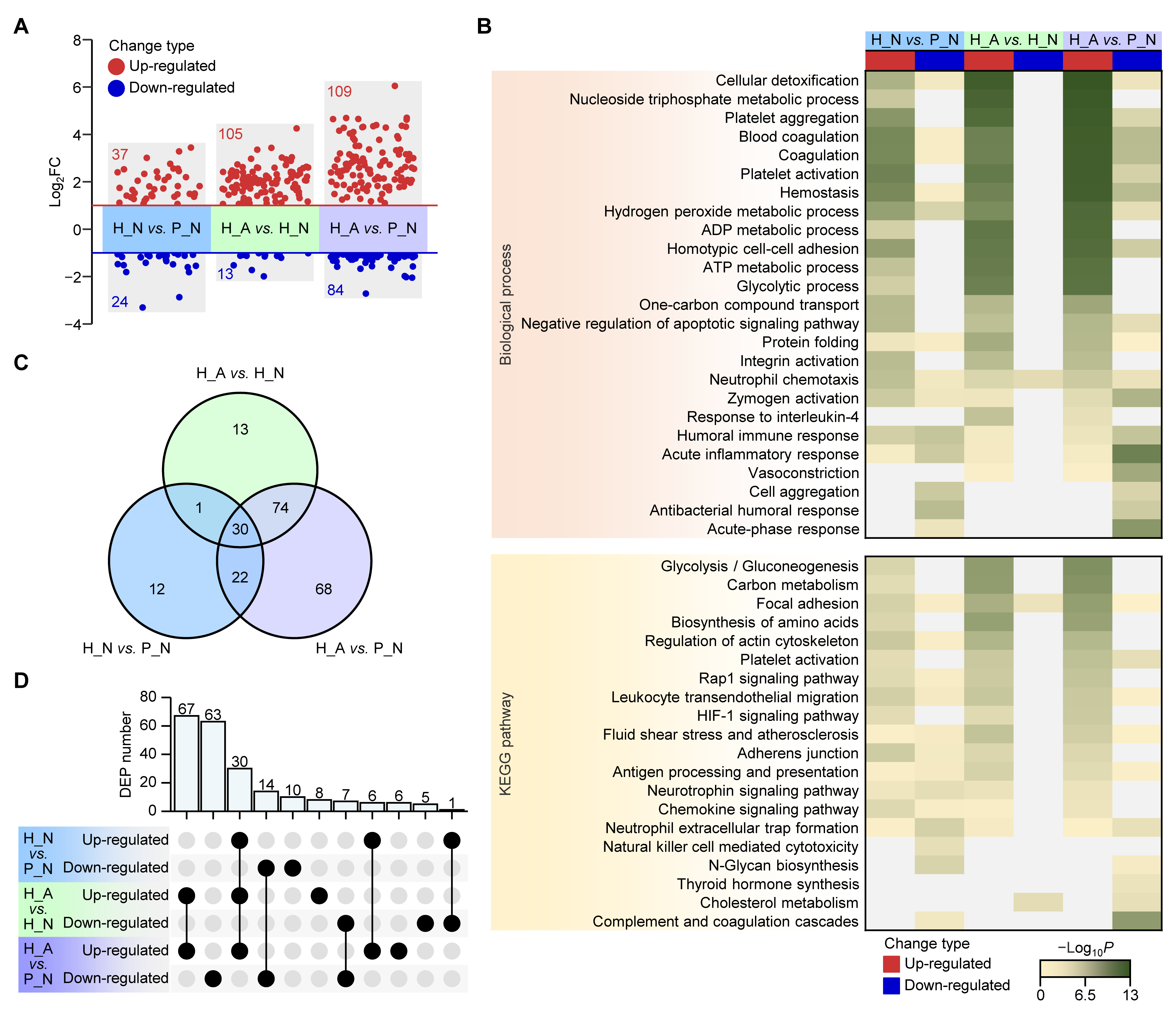

Supplement: qzaf030_Supplementary_Data [file qzaf030_supplementary_data.zip › Figure S2.tif]

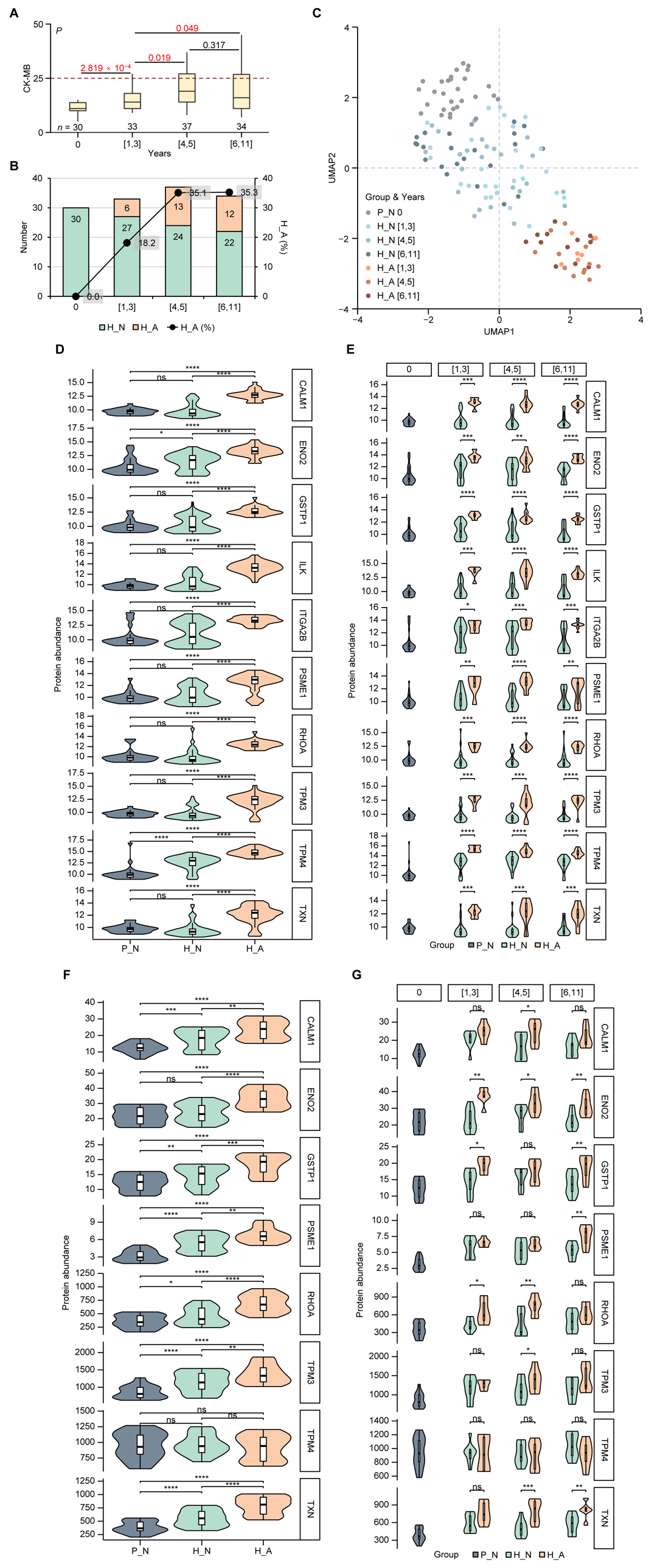

Supplement: qzaf030_Supplementary_Data [file qzaf030_supplementary_data.zip › Figure S3.tif]

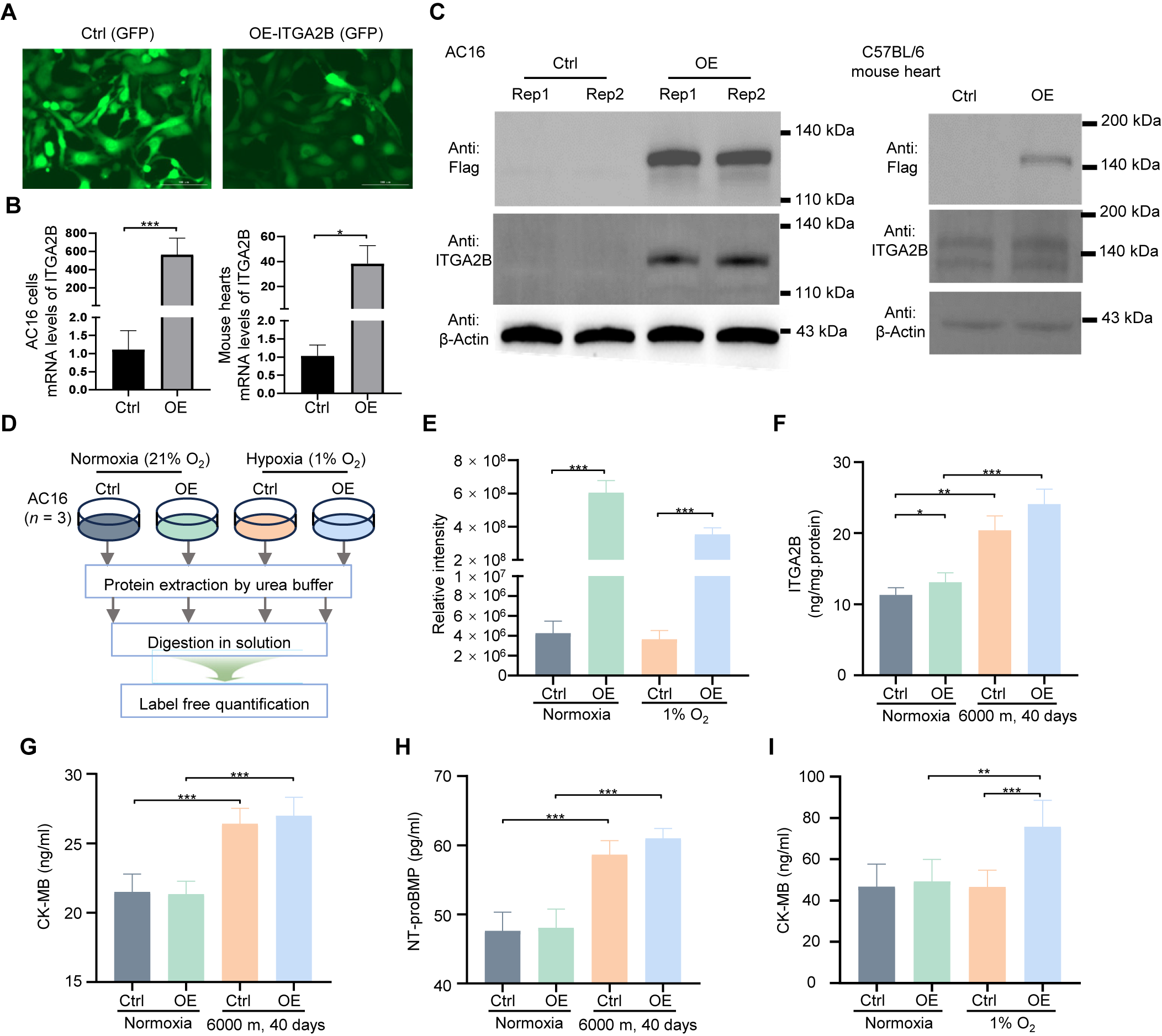

Supplement: qzaf030_Supplementary_Data [file qzaf030_supplementary_data.zip › Figure S4.tif]

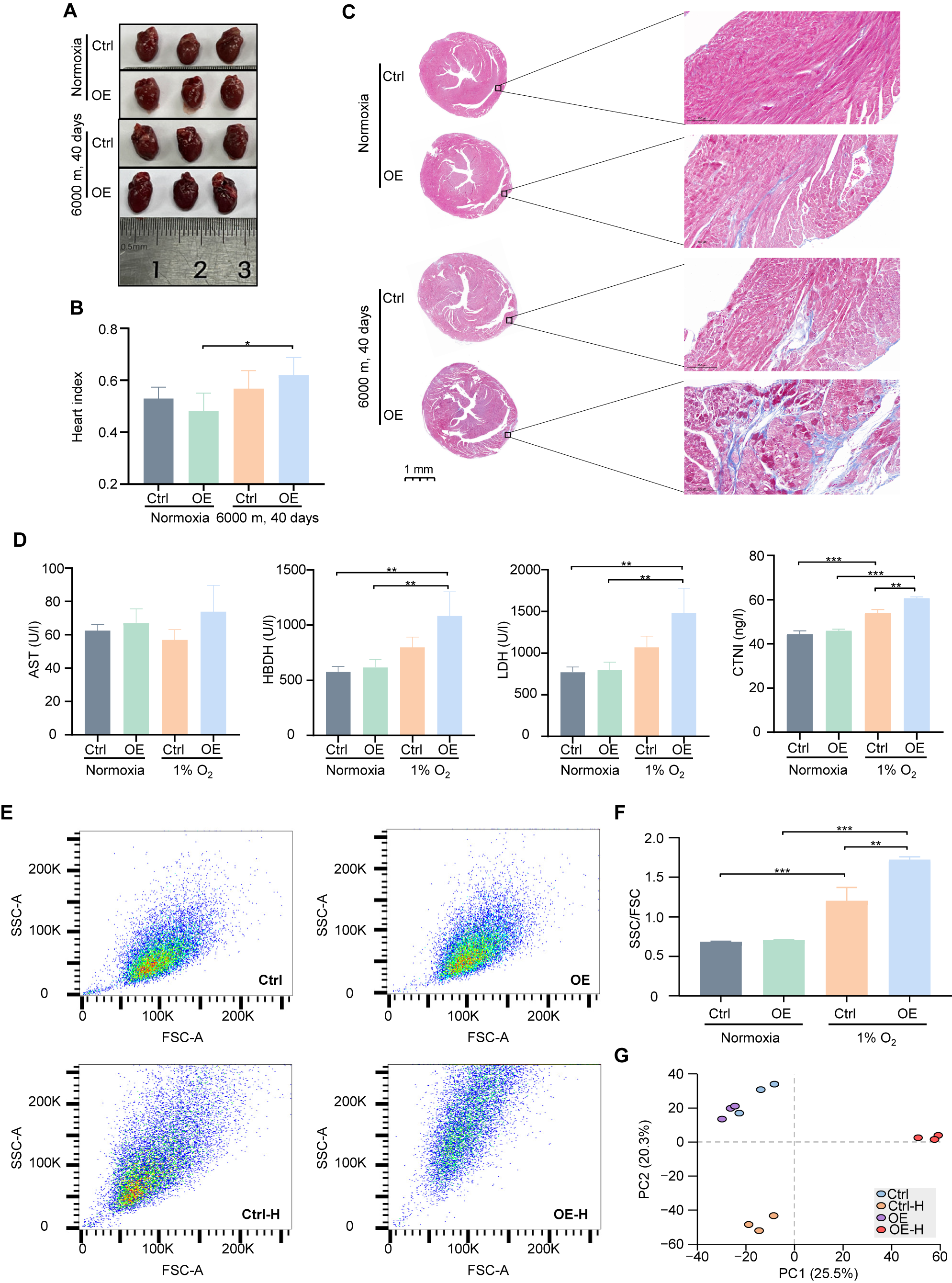

Supplement: qzaf030_Supplementary_Data [file qzaf030_supplementary_data.zip › Figure S5.tif]

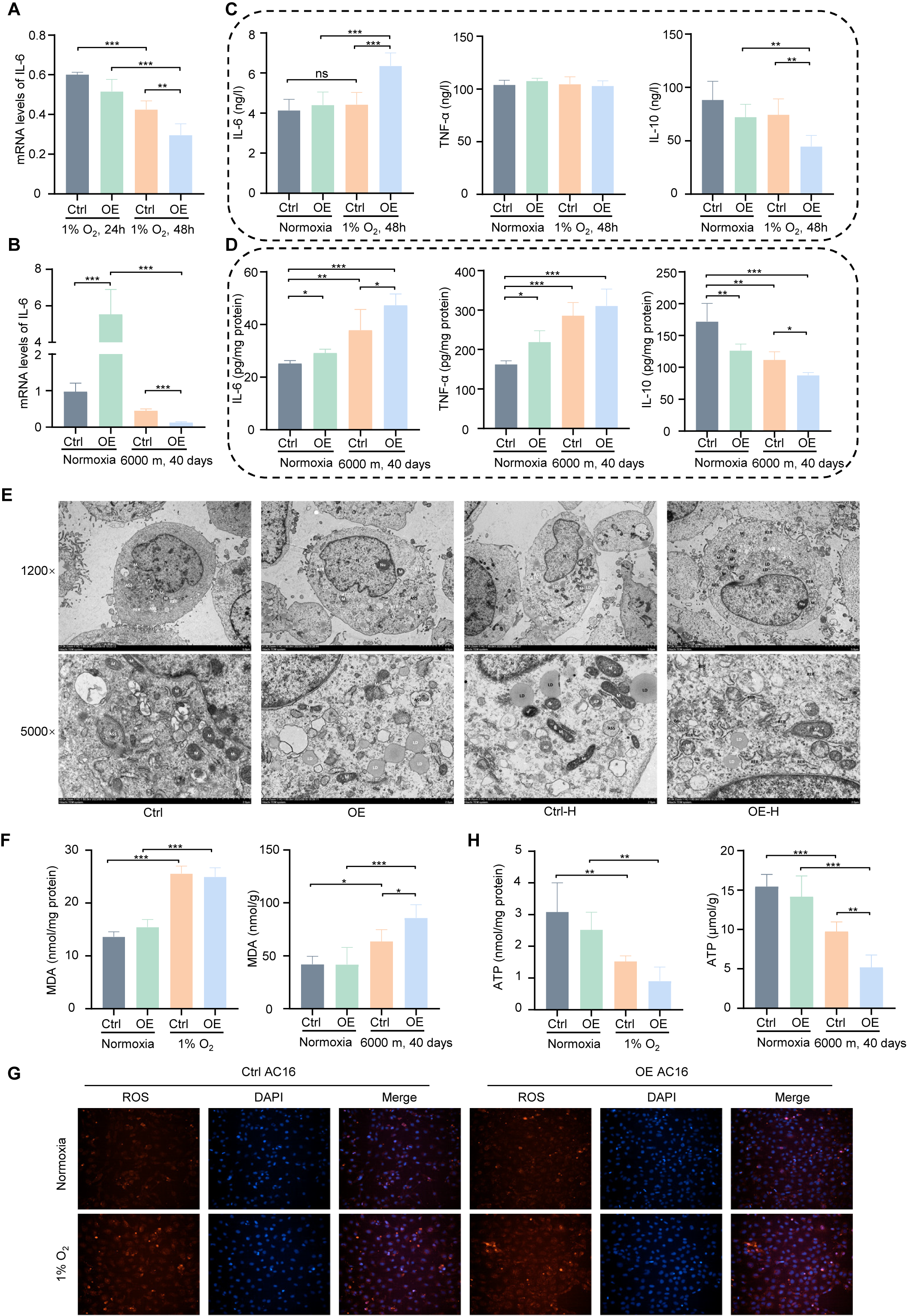

Supplement: qzaf030_Supplementary_Data [file qzaf030_supplementary_data.zip › Figure S6.tif]

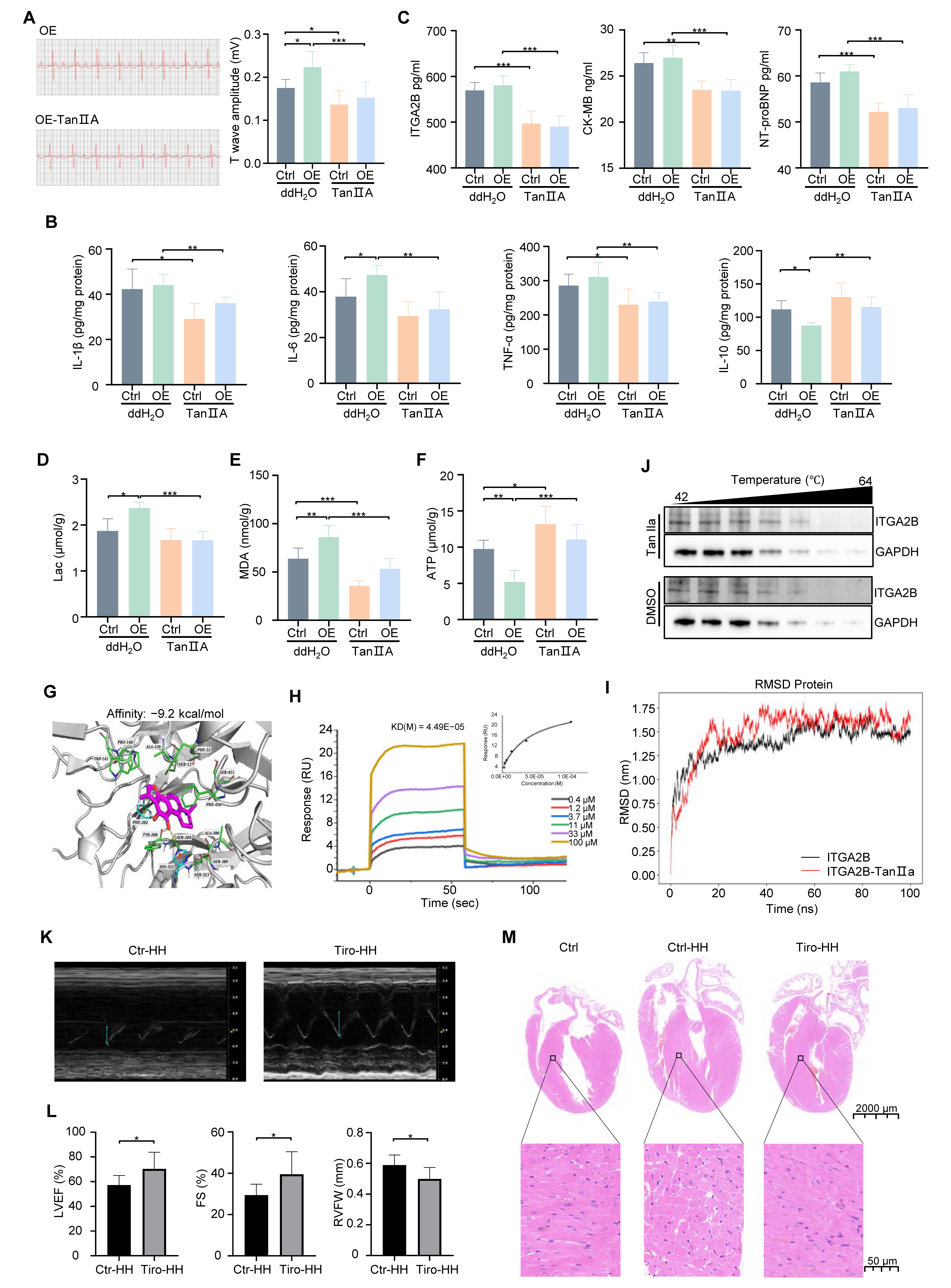

Supplement: qzaf030_Supplementary_Data [file qzaf030_supplementary_data.zip › Figure S7.tif]
